# Critical Review on Crystal Orientation Engineering of Antimony Chalcogenide Thin Film for Solar Cell Applications

**DOI:** 10.1002/advs.202304963

**Published:** 2023-11-08

**Authors:** Ke Li, Rongfeng Tang, Changfei Zhu, Tao Chen

**Affiliations:** ^1^ Hefei National Research Center for Physical Sciences at the Microscale CAS Key Laboratory of Materials for Energy Conversion Department of Materials Science and Engineering School of Chemistry and Materials Science University of Science and Technology of China Hefei Anhui 230026 P. R. China; ^2^ Institute of Energy Hefei Comprehensive National Science Center Hefei 230041 P. R. China

**Keywords:** antimony chalcogenides, charge transport, crystal orientation, solar cells

## Abstract

The emerging antimony chalcogenide (Sb_2_(S_x_Se_1−x_)_3_, 0 ≤ *x* ≤ 1) semiconductors are featured as quasi‐1D structures comprising (Sb_4_S(e)_6_)_n_ ribbons, this structural characteristic generates facet‐dependent properties such as directional charge transfer and trap states. In terms of carrier transport, proper control over the crystal nucleation and growth conditions can promote preferentially oriented growth of favorable crystal planes, thus enabling efficient electron transport along (Sb_4_S(e)_6_)_n_ ribbons. Furthermore, an in‐depth understanding of the origin and impact of the crystal orientation of Sb_2_(S_x_Se_1−x_)_3_ films on the performance of corresponding photovoltaic devices is expected to lead to a breakthrough in power conversion efficiency. In fact, there are many studies on the orientation control of Sb_2_(S_x_Se_1−x_)_3_ colloidal nanomaterials. However, the synthesis of Sb_2_(S_x_Se_1−x_)_3_ thin films with controlled facets has recently been a focus in optoelectronic device applications. This work summarizes methodologies that are applied in the fabrication of preferentially oriented Sb_2_(S_x_Se_1−x_)_3_ films, including treatment strategies developed for crystal orientation engineering in each process. The mechanisms in the orientation control are thoroughly analyzed. An outlook on perspectives for the future development of Sb_2_(S_x_Se_1−x_)_3_ solar cells based on recent research and issues on orientation control is finally provided.

## Graphical abstract

Crystal orientation engineering is critical for regulating the crystal plane‐dependent properties of quasi‐1D antimony chalcogenide materials, which in turn affects device efficiency. The regulation of crystal orientation is influenced by various factors such as growth rate, posttreatment, substrate type, interfacial engineering, and the introduction of seeding materials.

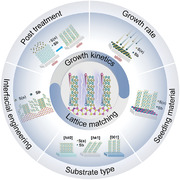

## 1 Introduction

Antimony chalcogenides (Sb_2_(S_x_Se_1−x_)_3_, 0 ≤ *x* ≤ 1), including antimony sulfide (Sb_2_S_3_), antimony selenide (Sb_2_Se_3_), and antimony selenosulfide (Sb_2_(S,Se)_3_), are widely used as light‐harvesting materials in solar cells due to their low toxicity, excellent chemical stability, and high light absorption coefficients (>10^5^ cm^−1^ in visible region). The recently developed hydrothermal deposition synthesis of Sb_2_(S_x_Se_1−x_)_3_ thin film has enabled the power conversion efficiency (PCE) of solar cells to exceed 10.7%, indicating its great potential in practical applications with further efficiency improvement.^[^ ^1^, ^2^ ^]^ Sb_2_(S_x_Se_1−x_)_3_ consists of 1D (Sb_4_S(e)_6_)_n_ ribbons, in which Sb─Se is covalently bonded along the c‐axis, while the ribbons interact with each other through weak van der Waals (vdW) force.^[^ ^3^ ^]^ This phenomenon leads to an anisotropic carrier transport property within the Sb_2_(S_x_Se_1−x_)_3_ films. Carrier transport along ribbons shows high efficiency, but hopping transport across ribbons appears very difficult. The maximum conductivity ratio between the c‐axis and vdW orientations is ≈16. Furthermore, the optical responsiveness ratio between these orientations is estimated to be 15, indicating that the carrier transport in the c‐axis orientation is highly effective.^[^ ^4^, ^5^ ^]^ The termination end of the (*hk*0) planes in the (Sb_4_S(e)_6_)_n_ ribbons does not necessarily result in the Sb─See bond breakage, thereby essentially decreasing the generation of dangling bonds. This characteristic results in [*hk*1]‐oriented Sb_2_(S_x_Se_1−x_)_3_ films with intrinsically benign grain boundaries that minimize recombination losses, displaying an advantage of quasi‐1D (Q1D) structured materials.^[^ ^6^ ^]^ Both ttheoretical study and extensive experimental investigations show that almost all high‐efficiency Sb_2_(S_x_Se_1−x_)_3_ solar cells have a high percentage of (Sb_4_S(e)_6_)_n_ ribbons that are inclined vertically to the substrate.^[^ ^6^, ^7^, ^8^, ^9^, ^10^ ^]^ For low‐dimensional materials, the surface energy of the crystalline plane parallel to the van der Waals force is always lower than that along the covalent bond direction.^[^ ^8^ ^]^ In terms of energy minimization principle, the (Sb_4_S(e)_6_)_n_ ribbons tend to grow parallel to the substrate to minimize the surface energy,^[^ ^11^ ^]^ making it very challenging to manipulate the growth of [*hk*1]‐oriented (Sb_4_S(e)_6_)_n_ ribbons.

Crystal orientation engineering generally refers to the regulation of the nucleation and growth behavior to enable the inorganic compound films to grow along specific crystal planes. Various methods are currently being used to prepare [*hk*1]‐oriented Sb_2_(S_x_Se_1−x_)_3_ films, which are generally solution‐based and vacuum deposition methods. The solution method mainly includes hydrothermal, chemical bath deposition, and spin‐coating methods, while the vacuum method primarily consists of rapid thermal evaporation, close space sublimation, vapor transport deposition, thermal evaporation, and magnetron sputtering deposition approaches.

The presence of different [*hk*1] film orientations indicates that the (Sb_4_S(e)_6_)_n_ ribbons tilt at different angles to the substrate. Ribbons with a low‐index facet specifically exhibit larger angles to the substrate and tend to grow perpendicular to it, while those with a high‐index facet indicate smaller ribbon angles to the substrate and tend to grow parallel to it. In films, the ribbon tilt angles to the substrate affect the carrier transport efficiency to a great extent. The [001]‐oriented ribbons perpendicular to the substrate particularly exhibit the best carrier transport performance due to their shorter transport distances compared to other [*hk*1]‐oriented ribbons.^[^ ^12^, ^13^, ^14^, ^15^ ^]^ The electron diffusion length in the [001]‐oriented (Sb_4_Se_6_)_n_ ribbons is approximately five times longer than that in the [221]‐oriented ribbons; thus, the [001] orientation is more conducive to improving the device performance.^[^ ^16^ ^]^ In addition, [001]‐oriented films have a higher growth rate along the ribbon direction, such that when the lateral strain generated between the (Sb_4_S(e)_6_)_n_ ribbons exceeds the tolerance range of the van der Waals forces during deposition, the films tend to transform into a nanorod array, which is a common structure in [001]‐oriented films.^[^ ^17^ ^]^ The [001]‐oriented nanorod array structured films reduce reflectivity and enhance light harvesting, which is beneficial to the performance of photovoltaic devices.^[^ ^18^ ^]^ This structure has been applied to solar cells and achieved breakthroughs in the PCE.^[^ ^17^, ^19^ ^]^ However, the nanorod array structure tends to form rough surface morphology, which leads to the generation of current leakage channels bringing about adverse effects in the open‐circuit voltage (*V*_OC_) and the fill factor (FF) of planar‐type solar cells.^[^ ^20^, ^21^ ^]^ Therefore, regulating the growth of ribbons to maintain an appropriate tilt angle is more suitable for application in planar heterojunction solar cell devices. Enhancing the lateral growth of ribbons to increase the film compactness and grain size is a key issue when developing [001]‐oriented structured devices. The presence of defects, such as dislocations related to the strain generated during the grain growth in the Sb_2_(S_x_Se_1−x_)_3_ films can inhibit the carrier transport along the ribbons. Therefore, developing advanced preparation methods that can better control the strain during the film growth process to fundamentally suppress the dislocation generation may be a promising direction.^[^ ^22^ ^]^

**Figure 1:**
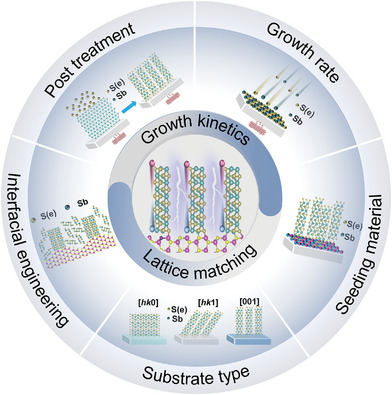
Schematic diagram of the currently developed strategies for regulating the Sb_2_(S_x_Se_1−x_)_3_ crystal orientation.

Controlling crystal orientation is a great challenge in the field of film preparation, and the application of advanced characterization techniques as well as understanding of the underlying mechanisms are essential for the study of crystal orientation.^[^ ^23^ ^]^ In this review, we first introduce common characterization methods that are used to quantitatively analyze the crystal orientation, including pole figure, orientation distribution map, texture coefficient, and ribbon carrier transport factors. Afterward, we summarize the relevant progress of the Sb_2_(S_x_Se_1−x_)_3_ crystal orientation engineering in terms of the orientation regulation methods and mechanisms, mainly analyzing the influence of film growth kinetics and interface lattice matching on the crystal growth orientation. We also discuss the orientation mechanisms of the Sb_2_(S_x_Se_1−x_)_3_ films from five aspects including growth rate, posttreatment, substrate type, interfacial engineering, and seeding material, as shown in **Figure** **1**. Lastly, we provide a brief outlook on the current challenges in crystal orientation engineering of Sb_2_(S_x_Se_1−x_)_3_ film for solar cell applications.

## 2 Crystallographic Orientation Characterization Methods

Theoretically, the orientation of each grain in a polycrystalline material is completely disordered and randomly distributed. However, the actual growth process always exhibits an inhomogeneously oriented crystal distribution, i.e., the growth orientation in certain directions significantly increases. This phenomenon is usually considered the preferred orientation. The orientation characteristics of polycrystalline films are described by the crystal texture, while the crystal texture existence indicates that the crystal has anisotropy,^[^ ^24^ ^]^ leading to significant differences in the carrier transport properties between [*hk*1]‐ and [*hk*0]‐oriented Q1D‐Sb_2_(S_x_Se_1−x_)_3_ films. The pole figure and texture coefficient based on X‐ray diffraction (XRD), as well as the orientation distribution map based on electron backscatter diffraction (EBSD), are useful techniques to analyze the crystal orientation. These analysis methods obtain information about the crystal structure and orientation from macroscopic at localized regions.^[^ ^25^ ^]^ We will briefly introduce herein the relevant characterization methods used to determine the crystal orientation.

### 2.1 Pole Figure

**Figure 2:**
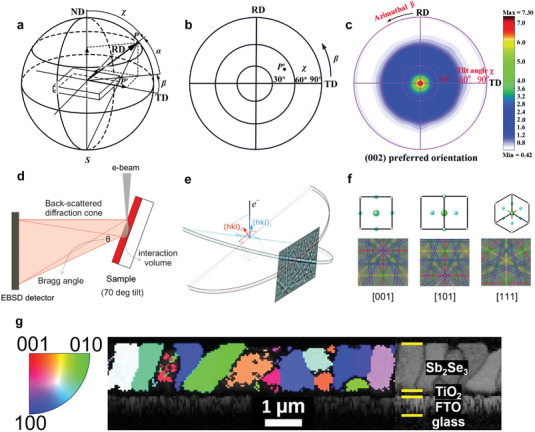
a) Schematic diagram of the pole figure measured by X‐ray goniometer. b) Pole figure obtained from 2D mapping of the pole density onto the equatorial plane. Adapted with permission.^[^ ^26^ ^]^ Copyright 2019, International Union of Crystallography. c) XRD pole figure of (002) plane preferred orientation, radial lines (purple) in the pole figure represent the tilt angle *χ* with an increment of 30°. Reproduced with permission.^[^ ^12^ ^]^ Copyright 2019, the Royal Society of Chemistry. d) Schematic of EBSD setup. Reproduced with permission under the terms of the Creative Commons CC‐BY‐NC license.^[^ ^31^ ^]^ Copyright 2018, Adhyaksa et al., Wiley‐VCH. e) Formation of Kikuchi patterns. Reproduced with permission.^[^ ^32^ ^]^ Copyright 2015, International Union of Crystallography. f) Simulated symmetry patterns for crystals at different orientations. Reproduced with permission under the terms of the Creative Commons CC‐BY license.^[^ ^25^ ^]^ Copyright 2020, Sun et al., Wiley‐VCH. g) EBSD map with orientation distribution of the Sb_2_Se_3_ film. Reproduced with permission under the terms of the Creative Commons CC‐BY‐NC‐ND license.^[^ ^15^ ^]^ Copyright 2021, Krautmann et al., Elsevier.

The pole figure is an important means of analyzing and determining the polycrystalline film texture, it is derived from the XRD characterization. Plotting the intensity of each (*hkl*) line relative to the sample coordinates in a stereographic projection enables us to qualitatively understand the crystallite orientation relative to the sample direction. These stereographic projection plots are defined as pole figures.^[^ ^26^ ^]^ In **Figure** **2**a, the sample's direction is defined by the transverse (TD), rolling (RD), and normal (ND) directions, respectively. The pole direction is defined by the radial *α*, azimuthal *β* angles, and tilt angle *χ*. The pole density of point *P* is defined as the point *P*′ projected by a straight line from point *P* to point *S* on the equatorial plane. The pole density in all directions can be mapped onto the equatorial plane through stereographic projection (Figure 2b). This method was recently used to analyze the Sb_2_(S_x_Se_1−x_)_3_ film orientation.^[^ ^12^, ^15^, ^27^, ^28^ ^]^ Zhou et al. implemented the XRD pole figure to demonstrate that the grains of Sb_2_Se_3_ films prepared by selenizing [003]‐oriented Sb films are mainly [001] preferred orientation.^[^ ^12^ ^]^ In Figure 2c, the XRD pole figure of the (002) plane shows a sharp single pole with a very narrow tilt angle of 10°, providing strong evidence for the highly preferred [001] orientation of the Sb_2_Se_3_ film.

### 2.2 Orientation Distribution Map

Electron backscatter diffraction (EBSD) is a scanning electron microscopy (SEM) based characterization technique commonly used to investigate the spatially resolved microstructural–crystallographic information of crystalline or polycrystalline materials with a sub‐micrometer resolution.^[^ ^29^ ^]^ Standard EBSD measurements are performed in the SEM chamber using either a direct electron detector or a conventional fluoroscope and camera (Figure 2d).^[^ ^25^ ^]^ A direct detector or phosphor screen very close to the sample allows imaging of backscattered electrons escaping from the sample at a specific Bragg angle, thus determining the orientation of the localized crystal. The resulting diffraction pattern is often referred to as a Kikuchi pattern, and diffraction of the lattice planes results in a series of intersecting bands called Kikuchi lines (Figure 2e). Since the Kikuchi bands correspond to the lattice planes, the angle between the bands can be utilized to determine the orientation of the crystal (Figure 2f).^[^ ^30^ ^]^ Scanning the beam over the surface of the sample collects patterns pixel by pixel, which can be indexed by comparing them with the simulated patterns, and ultimately results in a microstructure map of the sample, which includes information on crystal orientation, phase, strain, and grain boundaries. As shown in Figure 2g, the Sb_2_Se_3_ film exhibits randomly oriented grain distribution.^[^ ^15^ ^]^

### 2.3 Texture Coefficient

The texture coefficient (*TC*) is a commonly used parameter for quantitatively evaluating the preferred crystal plane orientation. The calculation formula for the *TC* of crystal planes (*hkl*) is as follows: $TC_{\left(\mathrm{hkl}\right)}=\frac{I_{\left(\mathrm{hkl}\right)}/I_{r(\mathrm{hkl})}}{\left[\left(1/n\right)\sum I_{\left(\mathrm{hkl}\right)}/I_{r(\mathrm{hkl})}\right]}$, where *I*_(_ *_hkl_*_)_, *I*_r(_ *_hkl_*_)_, and *n* indicate the intensity obtained from the XRD, intensity referring to the diffraction patterns from the inorganic crystal structure database (ICSD), and the number of diffraction peaks considered, respectively. The calculated *TC* ≈ 1 for all (*hkl*) crystal planes (*TC*_(_ *_hkl_*_)_) indicates that the film comprises randomly oriented grains similar to the information reflected by the ICSD. By contrast, *TC*_(_ *_hkl_*_)_ > 1 signifies that the film grows along the given (*hkl*) crystal plane as the preferred orientation, while *TC*_(_ *_hkl_*_)_ < 1 denotes that the orientation along the given (*hkl*) crystal plane is suppressed. Therefore, the *TC*_(_ *_hkl_*_)_ increase means the film tends to grow along the (*hkl*)‐preferred orientation.^[^ ^33^ ^]^

### 2.4 Ribbon Carrier Transport Factor

**Figure 3:**
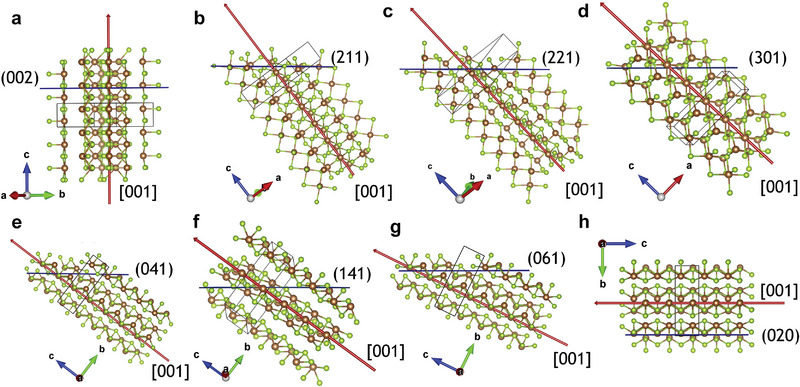
a–h) Graphical representations of the (Sb_4_Se_6_)_n_ ribbon inclination (red arrow, Sb^3+^ = brown dots, Se^2+^ = green dots) corresponding to [001] with respect to the growing surface (blue line) under different grain orientations. Reproduced with permission.^[^ ^27^ ^]^ Copyright 2020, Elsevier.

The *TC* is a convincing indicator for evaluating the films’ crystal orientation. It can also reflect the transport characteristics of carriers for polycrystalline materials. However, films usually contain multiple crystal planes with different [*hk*1] and [*hk*0] orientations. Pattini et al. calculated the ribbon carrier transport (*RCT*) factors for different oriented ribbons to quantitatively evaluate the contribution of different oriented ribbons to the effective carrier transport. The *RCT* is calculated according to *RCT*  =  *TC* × *EVC*, where *EVC* represents the component of the [*hkl*]‐oriented ribbons along the [001] direction. The (Sb_4_S(e)_6_)_n_ ribbons in Sb_2_(S_x_Se_1−x_)_3_ are stacked along the c‐axis direction, thereby requiring the ribbons to grow perpendicular to the substrate (i.e., along the [001] direction) to ensure a high‐efficiency carrier transport. The carriers show the worst transport performance when the ribbons are parallel to the substrate (i.e., along the [*hk*0] direction). The ribbons in the Sb_2_(S_x_Se_1−x_)_3_ film typically exhibit different orientations, with their angles ranging from 0° to 90° between the ribbons and the surface normal of the film. **Table** **1** lists the most common angles between the ribbons and the surface normal along with the effective vertical component of the ribbons defined by the strip angle cosine ranging from 0 ([*hk*0]‐oriented ribbon) to 1 ([001]‐oriented ribbon). **Figure** **3** depicts the corresponding crystal planes.^[^ ^27^, ^28^ ^]^

**Table 1:** Angles between the (Sb_4_S(e)_6_)_n_ ribbons and the surface normal and the effective vertical component of the ribbon orientation. Reproduced with permission.^[^ ^27^ ^]^ Copyright 2020, Elsevier.

Crystallographic plane	Angle between the ribbons and the surface normal [°]	Effective vertical component [EVC]
(002)	0	1
(211)	37.3	0.79
(221)	43.8	0.72
(301)	45.7	0.70
(041)	53.4	0.60
(141)	54.2	0.58
(061)	63.6	0.44
(*hk*0)	90	0

## 3 Orientation Control Strategies in Different Deposition Methods

Researchers exploit various deposition methods to prepare Sb_2_(S_x_Se_1−x_)_3_ films with enhanced [*hk*1] orientation. These methods are mainly classified into two: solution‐processed (e.g., hydrothermal and chemical bath deposition and spin‐coating method) and vacuum deposition (e.g., rapid thermal evaporation, close space sublimation, vapor transport deposition, thermal evaporation, and magnetron sputtering method) methods. This section presents an overview of each deposition method mentioned above and summarizes the approaches for enhancing the [*hk*1] orientation of the Sb_2_(S_x_Se_1−x_)_3_ films.

### 3.1 Hydrothermal Deposition

**Figure 4:**
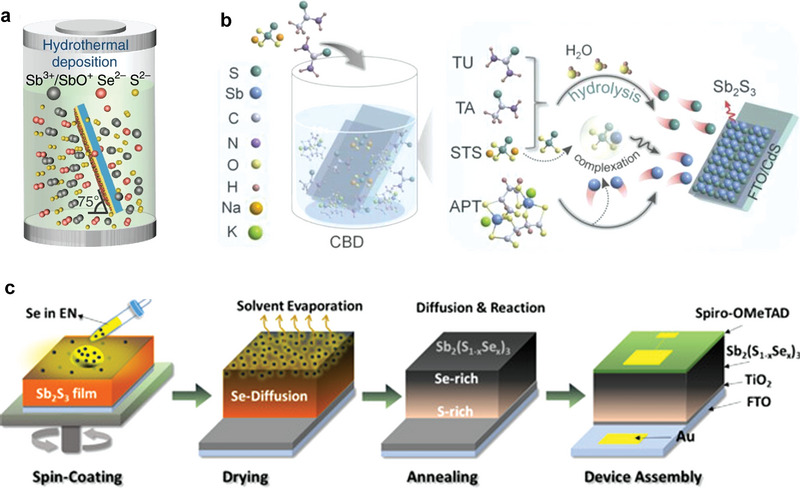
a) Schematic of the hydrothermal deposition of Sb_2_(S_x_Se_1−x_)_3_ in an autoclave, wherein KSbC_4_H_4_O_7_, Na_2_S_2_O_3_, and selenourea are employed as the Sb, S, and Se sources, respectively. Reproduced with permission.^[^ ^36^ ^]^ Copyright 2020, Springer Nature. b) Schematic illustration of the CBD process based on different sulfur sources. Reproduced with permission.^[^ ^48^ ^]^ Copyright 2022, Wiley‐VCH. c) Schematic illustration of the Sb_2_(S_x_Se_1−x_)_3_ film fabrication by spin coating. Reproduced with permission.^[^ ^60^ ^]^ Copyright 2017, Wiley‐VCH.

Hydrothermal deposition is an effective method of using an aqueous solution as the reaction system in a closed reactor (autoclave) to dissolve and recrystallize normally insoluble or poorly soluble substances by heating and pressurizing the reaction system (or autogenous vapor pressure), thereby creating a relatively high‐temperature and ‐pressure reaction environment for completing the inorganic material synthesis and treatment. This method is featured as a simple operation with environmental friendliness and low organic residues.^[^ ^34^, ^35^ ^]^ Sb_2_(S_x_Se_1−x_)_3_ prepared through this method shows an improved film quality with flat and compact surface morphology, increased grain size, and reduced defects, which enable the currently highest efficiency of Sb_2_(S_x_Se_1−x_)_3_ solar cells.^[^ ^1^, ^2^, ^36^, ^37^ ^]^ The hydrothermal deposition of the Sb_2_(S_x_Se_1−x_)_3_ film generally uses antimony potassium tartrate (KSbC_4_H_4_O_7_·0.5H_2_O) as the Sb source, sodium thiosulfate (Na_2_S_2_O_3_) as the S source, and selenourea and sodium selenosulfate (Na_2_SeSO_3_) as the Se sources. **Figure** **4**a presents a schematic of the hydrothermal deposition.

Several kinds of strategies have been developed to improve the [*hk*1] orientation of hydrothermally deposited Sb_2_(S_x_Se_1−x_)_3_ films. It has been observed that introducing additives (e.g., selenourea, thiourea, and ethanol) to the hydrothermal precursor solution is able to regulate the growth rate of Sb_2_(S_x_Se_1−x_)_3_ films,^[^ ^2^, ^36^, ^38^ ^]^ the presence of inorganic salts (e.g., Cd^2+^, F^−^, and Cl^−^ ions) facilitate the regulation of exposed crystalline planes of a substrate^[^ ^11^, ^39^, ^40^, ^41^, ^42^, ^43^ ^]^ and ultimately direct the oriented growth of Sb_2_(S_x_Se_1−x_)_3_ films. We will discuss the detailed mechanisms in Sections 4.1 and 4.4.

### 3.2 Chemical Bath Deposition

The chemical bath deposition (CBD) method is a solution reaction conducted at a mild temperature (<100 °C) and with atmospheric pressure. The ion release rate in the solution during this process is controlled by a complexation reaction. A chemical reaction occurs when the product of the anion and cation concentrations reaches the solubility product of the two ion types. Figure 4b depicts a schematic of the chemical reaction. This method possesses simple operation, low cost, and high capacity features.^[^ ^44^ ^]^ The reaction condition of the CBD is mild, allowing for better control of the reaction rate and the nucleation process. The CBD method is also beneficial to the formation of a relatively smooth and uniform film morphology.^[^ ^45^ ^]^ Reactants can also be timely added in situ during the reaction process, further increasing the flexibility in the film deposition.^[^ ^46^ ^]^ In 2014, the CBD achieved a 7.5% PCE in the Sb_2_S_3_ solar cells, indicating its potential for preparing metal chalcogenide films.^[^ ^47^ ^]^ In recent years, the CBD has made significant contributions to the Sb_2_(S_x_Se_1−x_)_3_ solar cell development. By adding different sulfur (i.e., Na_2_S_2_O_3_ and thioacetamide) and selenium (Na_2_SeSO_3_ and selenourea) sources to the CBD precursor solutions to deposit Sb_2_S_3_ and Sb_2_Se_3_, respectively, the film growth rate and [*hk*1] orientation have been improved, eventually obtaining the highest efficiencies for both the Sb_2_S_3_ and Sb_2_Se_3_ solar cells.^[^ ^45^, ^48^ ^]^

### 3.3 Spin‐Coating Method

Spin coating is a kind of convenient thin film fabrication method utilizing simple spin‐coater equipment. In film fabrication, a molecular precursor solution of the compound is spin‐coated on a substrate, followed by low‐temperature heating to evaporate the solvent and solidify the film. Further annealing at high temperatures promotes the solid‐state reaction, crystallization, and film formation (Figure 4c).^[^ ^49^ ^]^ This film fabrication method has the advantages of simplicity, and convenience, while a major portion of the solution is spun out without utilization.^[^ ^50^ ^]^ Various precursor solution systems, such as hydrazine solution with dissolved Sb, S, and Se powders,^[^ ^51^ ^]^ butyldithiocarbamate system with dissolved Sb_2_O_3_ and Se powders,^[^ ^52^ ^]^ and mixed solvents of *N*, *N*‐dimethylformamide (DMF) and dimethyl sulfoxide (DMSO) for dissolving SbCl_3_, thiourea, and selenourea to form a homogeneous solution, are currently being developed to prepare Sb_2_(S_x_Se_1−x_)_3_ films.^[^ ^53^ ^]^ Notably, by adding a tiny amount of water into the mixed DMF and DMSO solvent to dissolve the Sb, S, and Se precursors, Wu et al. prepared Sb_2_(S_x_Se_1‐x_)_3_ films with increased grain size and suppressed deep‐level defects and finally achieved a PCE of 7.42%.^[^ ^53^ ^]^ Various strategies based on spin coating have also been developed to regulate the growth habits of the Sb_2_(S_x_Se_1−x_)_3_ film to enhance its [*hk*1] orientation. In this regard, the [*hk*1]‐oriented Sb_2_Se_3_ nanorod arrays are prepared by dissolving SbCl_3_ and Se powder in the mixed solvent of thioglycolic acid, ethanolamine, and 2‐methoxyethanol. The formation mechanism of nanorod arrays is attributed to the adsorption of carboxylate anions as capping agents on the side ends of the (Sb_4_Se_6_)_n_ ribbons inhibiting the lateral growth of ribbons.^[^ ^54^, ^55^, ^56^ ^]^ The [*hk*1]‐oriented Sb_2_S_3_^[^ ^57^, ^58^ ^]^ and Sb_2_Se_3_^[^ ^59^ ^]^ nanorod arrays are obtained by spin‐coating a seeding material on the substrate. Further mechanistic discussions will be presented in Section 4.5.

### 3.4 Rapid Thermal Evaporation

**Figure 5:**
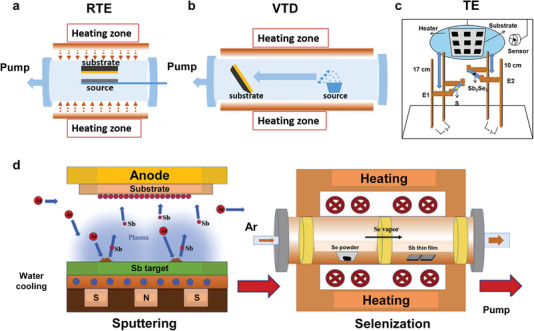
Schematic diagrams of different vacuum deposition methods: a) rapid thermal evaporation (RTE) method and b) vapor transport deposition (VTD) method. Reproduced with permission.^[^ ^75^ ^]^ Copyright 2020, Wiley‐VCH. c) Thermal evaporation (TE) method. Reproduced with permission.^[^ ^81^ ^]^ Copyright 2021, Wiley‐VCH. d) Radio frequency (RF) magnetron sputtering of the Sb film and the subsequent postselenization treatment. Reproduced with permission.^[^ ^88^ ^]^ Copyright 2020, Elsevier.

Sb_2_Se_3_ has a melting point of 608 °C with a high saturated vapor pressure of ≈1200 Pa at 550 °C, which enables Sb_2_Se_3_ films to be deposited at a high deposition rate through the vapor deposition method. The rapid thermal evaporation (RTE) method can control the deposition rate up to 1 µm min^−1^, which is much higher than that of the conventional thermal evaporation (typically 0.01–0.1 µm min^−1^) and magnetron sputtering (typically 0.01–0.05 µm min^−1^) methods. In 2015, Zhou et al. developed an RTE method for the Sb_2_Se_3_ film preparation. During this process, the Sb_2_Se_3_ powder can directly be evaporated under a low vacuum pressure of ≈8 mTorr and condensed onto the substrate at gradient temperature to form high‐quality Sb_2_Se_3_ film. The distance between the evaporation source and the substrate is controlled at 0.8 cm to achieve a fast deposition rate and a high material utilization ratio. **Figure** **5**a depicts a schematic of the RTE deposition process. Based on the RTE method, a significant [*hk*1]‐oriented growth of the Sb_2_Se_3_ film can be achieved through regulation strategies, including the exposure of the specific crystalline planes of the interfacial material^[^ ^9^, ^61^ ^]^ and the introduction of seeding materials.^[^ ^8^ ^]^

### 3.5 Close Space Sublimation

Similar to the RTE method, the close space sublimation (CSS) method has the characteristics of a high deposition rate and a close source–substrate distance. It is one of the promising technologies for large‐scale manufacturing.^[^ ^62^ ^]^ The only difference is that the CSS deposition is completed through solid sublimation and does not undergo the solid melting process like the RTE method.^[^ ^6^ ^]^ The CSS method is widely used in the CdTe solar cell preparation due to its excellent scalability, high throughput, and efficient material utilization.^[^ ^63^ ^]^ In recent years, it has also been used to prepare Sb_2_(S_x_Se_1−x_)_3_ films, exhibiting great potential in obtaining high‐efficiency solar cells. For the first time, Phillips et al.^[^ ^64^ ^]^ and Zeng et al.^[^ ^65^ ^]^ prepared Sb_2_Se_3_ and Sb_2_S_3_ films using the CSS method, while Li et al. prepared CSS‐deposited Sb_2_(S_x_Se_1‐x_)_3_ films by Sb_2_Se_3_ and Sb_2_S_3_ co‐sublimation.^[^ ^66^ ^]^

In terms of the orientation control and the solar cell efficiency improvement, selenization has been performed on Mo/W/Pb substrates to induce highly [001]‐oriented (Sb_4_Se_6_)_n_ ribbons. As a result, a significant improvement in the device's performance is achieved.^[^ ^7^, ^17^, ^19^, ^67^ ^]^ Some other interface regulation methods,^[^ ^68^ ^]^ together with the introduction of seeding materials,^[^ ^69^ ^]^ have also been proven effective in promoting the [*hk*1] orientation of Sb_2_(S_x_Se_1−x_)_3_ films.

### 3.6 Vapor Transport Deposition

Vapor transport deposition (VTD) is an excellent technique for the mass production of CdTe thin‐film solar cells and has been successfully employed in Sb_2_(S_x_Se_1−x_)_3_ solar cells. In the VTD, the distance between the substrate and the evaporation source is adjustable, allowing for an independent adjustment of the substrate temperature by changing the distance. Figure 5b is a schematic of the VTD. This feature can effectively reduce the inaccurate temperature control caused by the temperature coupling between the evaporation source and the substrate during the film deposition process, which is the problem faced by the RTE and CSS methods. The VTD process also increases the gas‐phase transport distance, which promotes uniform mixing of gas‐phase particles (e.g., Se, Sb, and Sb_x_Se_y_). A VTD device with a larger source–substrate distance will cause sulfur loss in CdS, which is more conducive to obtaining vertically oriented Sb_2_S_3_ films.^[^ ^70^ ^]^ Therefore, most of the high‐efficiency Sb_2_(S_x_Se_1−x_)_3_ solar cells prepared through the VTD method are based on the CdS substrates,^[^ ^71^, ^72^, ^73^, ^74^ ^]^ while those prepared through the RTE and CSS methods are mostly based on metal oxide or Mo substrates.^[^ ^7^, ^8^, ^9^, ^17^, ^19^, ^63^, ^69^ ^]^ In 2018, Wen et al. prepared highly oriented and improved‐crystallinity Sb_2_Se_3_ films using the VTD method, achieving a 7.6% PCE.^[^ ^74^ ^]^ Zhang et al. developed a vertical VTD (V‐VTD) method to enhance the [*hk*1] orientation of the Sb_2_(S_x_Se_1−x_)_3_ films prepared through the VTD method. Compared with the traditional VTD, the main improvement in the V‐VTD is the sufficient space between the substrate and the evaporation source that provides a relatively wide regulation range and achieves a preferential [*hk*1] growth orientation of Sb_2_S_3_.^[^ ^75^ ^]^ The orientation filtering^[^ ^18^ ^]^ and postselenization treatment^[^ ^76^ ^]^ strategies are more favorable to the growth of [*hk*1]‐oriented Sb_2_(S_x_Se_1‐x_)_3_ films. The detailed mechanisms are discussed in Sections 4.1.3 and 4.2.2.

### 3.7 Thermal Evaporation

Thermal evaporation is a common vacuum method for preparing thin films. In the deposition process, the raw materials are heated in an evaporation boat inside a vacuum chamber to form molecules or atoms, followed by escaping to the substrate surface through the stream flow and solidifying to form films (Figure 5c). The thermal evaporation method possesses the characteristics of easy operation, simple deposition mechanism, and high‐purity of the prepared films.^[^ ^77^ ^]^ Notably, the highly saturated vapor pressure of Se makes it prone to loss during the Sb_2_Se_3_ film preparation. Zhang et al. annealed thermally evaporated Sb_2_Se_3_ films under the H_2_Se and H_2_S atmospheres to compensate for the lost Se. However, the treated films transformed to an unfavorable [*hk*0] orientation.^[^ ^78^ ^]^ Li et al. compensated for the Se loss by coevaporating the Sb_2_Se_3_ and Se powders and demonstrated a beneficial effect on the preferred orientation of the deposited Sb_2_Se_3_ films.^[^ ^79^ ^]^ The effects of the thermal evaporation conditions, such as the substrate temperature,^[^ ^80^ ^]^ source–substrate distance, and coevaporation sequence,^[^ ^81^ ^]^ on the film orientation have been studied. Accordingly, some interface regulation methods have been found favorable for obtaining [*hk*1]‐oriented Sb_2_(S_x_Se_1−x_)_3_ films.^[^ ^82^ ^]^

### 3.8 Magnetron Sputtering Deposition

Magnetron sputtering is a method of ionizing Ar ions under an electric field to rapidly bombard the target and produce gas particles for deposition on a substrate. This method has the features of a large deposition area, fast deposition rate, easy control, and strong film adhesion. Magnetron sputtering is classified into direct current (DC) and radio frequency (RF) magnetron sputtering. In 2017, Liang et al. prepared [221]‐oriented Sb_2_Se_3_ nanorod array films through RF magnetron sputtering by sputtering a single Sb_2_Se_3_ target with a substrate temperature of 375 °C, achieving a 2.11% PCE.^[^ ^83^ ^]^ They also systematically studied the effect of the substrate temperature on the Sb_2_Se_3_ film orientation and demonstrated amorphous films prepared at a substrate temperature below 150 °C.^[^ ^84^ ^]^ Subsequently, they performed a postselenization treatment on the amorphous film to prepare an [*hk*1]‐oriented Sb_2_Se_3_ film, obtaining a 6.06% PCE.^[^ ^85^ ^]^ Numerous studies have shown that the postselenization of the prepared Sb thin films is beneficial in preparing Sb_2_Se_3_ films with [001]‐preferred orientation.^[^ ^12^, ^13^, ^86^, ^87^, ^88^, ^89^, ^90^ ^]^ Figure 5d depicts the schematic for this. In 2016, Yuan et al. deposited an Sb film on the Mo substrate through DC magnetron sputtering, followed by a postselenization to prepare the Sb_2_Se_3_ thin film, and applied it to the substrate‐structure solar cells. However, the device's efficiency was not high.^[^ ^91^ ^]^ Liang et al. deposited Sb thin films using the RF magnetron sputtering method. This was followed by a postselenization treatment that not only prepared [001]‐oriented Sb_2_Se_3_ films, but also improved the solar cell PCE to 6.84% with an open‐circuit voltage higher than 500 mV.^[^ ^88^ ^]^ Furthermore, Liang X et al.^[^ ^92^ ^]^ and Spaggiari et al.^[^ ^28^ ^]^ have verified the effects of the chamber pressure, substrate type, and substrate temperature on the orientation of magnetron‐sputtered Sb_2_Se_3_ films, consequently providing theoretical and experimental bases for the further improvement of the film orientation and the device efficiency in the future.

## 4 Mechanisms of the Orientation Regulation

The kinetic energy of the deposited particles has a modulating effect on the crystal structure, defects, and film orientation.^[^ ^93^, ^94^, ^95^, ^96^ ^]^ The kinetic energy of the particles can be evaluated using the mean free path (*λ*) defined as $\lambda \;=\frac{k_{B}T}{\sqrt{2}\pi d^{2}p}\;$, where *T* is the temperature; *d* is the molecular diameter; *p* is the pressure; and *k*_B_ is the Boltzmann constant.^[^ ^97^ ^]^ This equation shows that the evaporation source temperature and the reaction chamber pressure are both factors influencing the kinetic energy of particles. The evaporation source temperature can directly affect the evaporation rates, while the chamber pressure affects the deposition rate of particles reaching the substrate surface via collisions between vapor and chamber gas particles. In the practical deposition process, researchers tend to focus more on the film growth rate. The correlation between the growth and the deposition rates can be defined as the adhesion coefficient related to the substrate temperature, composition, and structure.^[^ ^98^ ^]^ In addition, the posttreatment also has a regulating effect on the film's oriented growth.

**Figure 6:**
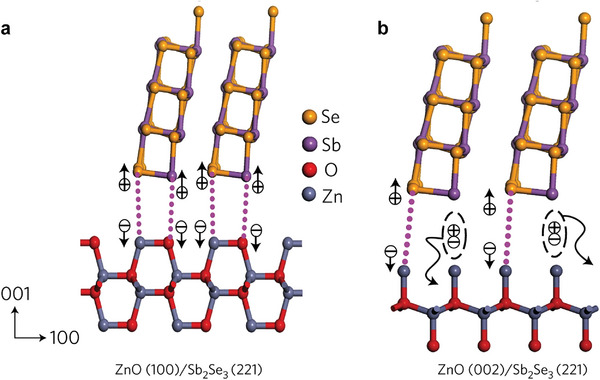
Atomistic model of the [221]‐oriented Sb_2_Se_3_ film on the (100) (a) and (002) (b) planes of ZnO. The dotted light magenta lines represent the bond formation leading to a successful charge separation at the interface. The absence of these lines indicates the generation of the dangling bonds causing a recombination loss. + represents the hole, while − represents the electron. The straight arrows represent a smooth carrier separation, while the curvilineal arrows represent the recombination loss. Reproduced with permission.^[^ ^9^ ^]^ Copyright 2017, Springer Nature.

The difference in the polar and nonpolar crystal plane characteristics allows the crystal surface to present completely different atomic stacking orders. For the crystal ZnO shown in **Figure** **6**, the Zn and O atoms are uniformly distributed on the nonpolar planes but alternately distributed on the polar ones.^[^ ^9^ ^]^ The different crystal planes exposed on the substrate result in different bonding modes between the (Sb_4_S(e)_6_)_n_ ribbons and the substrate, which played a crucial role in controlling the Sb_2_(S_x_Se_1−x_)_3_ film orientation. For the randomly oriented ZnO with exposed nonpolar (100) planes, the uniformly distributed Zn and O atoms on the surface can form Zn─Se and Sb─O bonds with Se and Sb at the ends of (Sb_4_Se_6_)_n_ ribbons, respectively. Thus, the (100)‐oriented ZnO plane can induce [221]‐oriented Sb_2_Se_3_ films (Figure 6a). In contrast, when the (002) polar plane of ZnO is exposed, only Zn or O atoms are exposed on the surface, thus forming only one type of bonding with the (Sb_4_S(e)_6_)_n_ ribbons. Either the Zn^2+^ end forms a Zn─Se bond with the exposed Se atom on the (001) planes of Sb_2_Se_3_ or the O^2−^ end forms a Sb─O bond with the exposed Sb atom (Figure 6b). This interfacial bonding causes a lattice mismatch and results in the generation of dangling bonds and poor adhesion at the interface, depicting a thermodynamic instability that influences the (Sb_4_Se_6_)_n_ ribbons to tend to grow along the [120] orientation.^[^ ^41^, ^73^ ^]^ Extensive studies developed a series of methods for improving the lattice matching degree between the interfacial material and the [*hk*1]‐oriented ribbons, including matching more suitable substrates, interfacial engineering, and introducing seeding materials.

This section summarizes the orientation control mechanisms of the Sb_2_(S_x_Se_1−x_)_3_ films prepared using different methods. **Table** **2** presents the deposition methods, regulation mechanism of the oriented films, preferred orientations, *TC*s, device configurations, and device performance (PCE) mentioned in this work.

**Table 2:** Summary of the device configurations, deposition methods, orientation mechanisms, preferred orientations, texture coefficients, and PCE of the Sb_2_(S_x_Se_1−x_)_3_ films and solar cell devices.

Device configuration^a)^	Deposition method^b)^	Orientation regulation mechanism	Preferred orientation	Texture coefficient	PCE [%]	Reference
FTO/CdS/Sb_2_(S,Se)_3_/Spiro‐OMeTAD/Au	HD	Growth rate	[221]	/	10.75	[2]
FTO/CdS/Sb_2_(S,Se)_3_/Spiro‐OMeTAD/Au	HD	Growth rate	[221]	/	10.1	[36]
FTO/CdS/Sb_2_Se_3_/Spiro‐OMeTAD/Au	HD	Growth rate	[211]/[221]	/	7.9	[38]
FTO/CdS/Sb_2_Se_3_/Spiro‐OMeTAD/Au	CBD	Growth rate	[221]/[301]	/	10.57	[45]
FTO/CdS/Sb_2_S_3_/Spiro‐OMeTAD/Au	CBD	Growth rate	[211]/[221]	/	8.0	[48]
FTO/CdS/Sb_2_Se_3_/Au	RTE	Growth rate	[221]	/	5.6	[6]
ITO/CdS/Sb_2_(S,Se)_3_/Au	VTD	Growth rate	[221]	/	7.6	[99]
ITO/CdS/Sb_2_Se_3_/Au	VTD	Growth rate	[221]	/	7.6	[100]
ITO/CdS/Sb_2_Se_3_/C/Ag	VTD	Growth rate	[221]	/	6.09	[101]
ITO/CdS/Sb_2_S_3_/Au	VTD	Growth rate	[211]/[221]	2.1	4.5	[102]
Mo/Sb_2_Se_3_/CdS/ZnO/AZO/Al	TE	Growth rate	[211]	2.5	4.5	[80]
FTO/CdS/Sb_2_(S,Se)_3_/PCBM/Ag	HD	Post‐treatment	[211]	/	9.22	[103]
FTO/CdS/Sb_2_S_3_/spiro‐OMeTAD/Au	HD	Post‐treatment	[211]/[221]	1.4	6.82	[104]
ITO/TiO_2_/CdS/Sb_2_S_3_/C/Ag	HD	Post‐treatment	[041]/[141]	5.5	7.23	[3]
Mo/Sb_2_S_3_/CdS/i‐ZnO/AZO/Ni/Al	PE + sulfuration	Post‐treatment	[111]	2.3	3.35	[10]
Mo/Sb_2_Se_3_/CdS/ITO/Ag	CSS + Selenization	Selenization kinetics	[001]	3.1	4.86	[20]
Mo/Sb_2_Se_3_/CdS/ITO/Ag	VTD + Selenization	Selenization kinetics	[101]/[001]	1.9	7.40	[76]
mica/Mo/Sb_2_Se_3_/CdS/ITO/Ag grid	TE + Selenization	Selenization kinetics	[001]	3.4	8.42	[86]
FTO/ZnO/Sb_2_Se_3_/Au	RTE	Lattice matching	[211]/[221]	/	6	[9]
FTO/TiO_2_/Sb_2_S_3_/Se‐treated/Au	RTE	Lattice matching	[221]	/	5.4	[61]
Ag/ITO/epi‐CdS/Sb_2_Se_3_/Au/SU‐8	VTD	Lattice matching	[322]/[422]/[041]	1.75	7.15	[14]
ITO/SnO_2_/TiO_2_/CdS/Sb_2_Se_3_/Au	VTD	Lattice matching	[041]/[141]	2.5	7.0	[105]
FTO/SnO_2_/CdS/Sb_2_(S,Se)_3_/Spiro‐OMeTAD/Au	HD	Interfacial engineering	[221]	/	9.37	[42]
FTO/SnO_2_/CdS/Sb_2_(S,Se)_3_/Spiro‐OMeTAD/Au	HD	Interfacial engineering	[101]/[111]	2.3	9.2	[41]
FTO/CdS:O/Sb_2_(S,Se)_3_/Spiro‐OMeTAD/Au	HD	Interfacial engineering	[211]/[221]/[301]	1.25	8.59	[106]
FTO/TiO_2_/Sb_2_(S,Se)_3_/Spiro‐OMeTAD/Au	HD	Interfacial engineering	[211]/[221]	1.4	7.08	[107]
ITO/CdS:In/Sb_2_S_3_/Spiro‐OMeTAD/Au	HD	Interfacial engineering	[211]/[221]	/	7.15	[43]
FTO/TiO_2_/CdS/Sb_2_S_3_/spiro‐OMeTAD/Au	HD	Interfacial engineering	[211]/[221]	/	6.4	[11]
FTO/CdS/Li‐Sb_2_S_3_/PbS/C	HD	Interfacial engineering	[211]/[221]	/	6.16	[40]
Mo/MoSe_2_/Sb_2_Se_3_/CdS/ZnO/AZO	IVD	Interfacial engineering	[001]	10	10.1	[19]
Mo/MoSe_2_/Sb_2_Se_3_/HR‐ZnO/LR‐ZnO/Ag	CSS	Interfacial engineering	[001]	/	9.2	[17]
W/WSe2/Sb_2_Se_3_/CdS/i‐ZnO/AZO	CSS	Interfacial engineering	[001]	/	8.46	[67]
PI/Mo/PbSe/ Sb2Se3/CdS/i‐ZnO/AZO/Ag	CSS	Interfacial engineering	[001]	2.8	8.43	[7]
FTO/CdS:O/Sb_2_Se_3_/Graphite/Ag	CSS	Interfacial engineering	[211]/[221]	1.5	6.3	[108]
FTO/SnO_2_/Sb_2_Se_3_/P3HT/C	CSS	Interfacial engineering	[001]	3.5	5.41	[68]
ITO//CdS/Sb_2_(S,Se)_3_/Au	VTD	Interfacial engineering	[221]	/	7.12	[73]
ITO/CdS/Sb_2_Se_3_/Spiro‐OMeTAD/Au	VTD	Interfacial engineering	[211]/[221]	/	7.48	[109]
ITO/TiO_2_/Sb_2_Se_3_/Au	VTD	Interfacial engineering	[221]/[301]/[141]	2	5.33	[110]
ITO/CdS/Sb_2_S_3_/Au	VTD	Interfacial engineering	[211]/[121]	/	4.73	[70]
FTO/CdS/Sb_2_(S,Se)_3_/Spiro‐OMeTAD/Au	TE	Interfacial engineering	[221]	/	8.0	[81]
FTO/CdS/Sb_2_Se_3_/spiro‐OMeTAD/Au	TE	Interfacial engineering	[221]	1.6	6.89	[82]
FTO/TiO_2_/Sb_2_S_3_/PTB7/MoO_3_/Au	Spin coating	Seeding material	[211]	/	7.18	[111]
FTO/TiO_2_/Sb_2_S_3_/PTB7/MoO_3_/Au	Spin coating	Seeding material	[211]	/	5.7	[57]
FTO/TiO_2_/Sb_2_S_3_/Spiro‐OMeTAD/Au	Spin coating	Seeding material	[221]	/	5.15	[58]
FTO/TiO_2_/Sb_2_Se_3_/PbS/Au	RTE	Seeding material	[211]/[221]	7	7.62	[8]
FTO/CdS/CeO_2_/Sb_2_Se_3_/Au	RTE	Seeding material	[211]/[221]	4	5.14	[112]
Mo/Sb_2_Se_3_/CdS/i‐ZnO/AZO/Ag grid	CSS	Seeding material	[211]/[221]/[001]	1.8	8.5	[69]
FTO/CdS/Sb_2_S_3_ seed/Sb_2_Se_3_/Spiro‐OMeTAD/C	CSS	Seeding material	[001]	1.2	7.44	[113]
FTO/CdS/Sb_2_Se_3_/Au	CSS	Seeding material	[221]	1.5	5.3	[114]
FTO/CdS/Al_2_O_3_/Sb_2_Se_3_/C	VTD	Seeding material	[101]/[211]/[221]	1.7	6.25	[115]
Mo/Sb_2_Se_3_/CdS/ITO/Ag	MS + selenization	Seeding material	[211]/[221]/[001]	/	8.14	[116]

^a)^ FTO, fluorine‐doped tin oxide; ITO, indium tin oxide; AZO, aluminum‐doped ZnO; PCBM, [6,6]‐phenyl‐C61‐butyric acid methylester; SU‐8, photoresist; HR‐ZnO, high‐resistance ZnO; LR‐ZnO, low‐resistance ZnO; PI, polyimide; P3HT, poly(3‐hexylthiophene); and PTB7, poly[4,8‐bis(5‐(2ethylhexyl) thiophen‐2‐yl)benzo[1,2‐b;4,5‐b′]dithiophene‐2,6diyl‐alt‐(4‐(2‐ethylhexyl)−3‐fluorothieno[3,4‐b]thiophene‐)−2‐carboxylate‐2‐6‐diyl)];

^b)^ HD, hydrothermal deposition; CBD, chemical bath deposition; RTE, rapid thermal evaporation; CSS, close space sublimation; VTD, vapor transport deposition; TE, thermal evaporation; MS, magnetron sputtering; and PE, pulse electrodeposition.

### 4.1 Growth Rate

The film growth rate is influenced by the deposition rate and the adhesion coefficient between the film and the substrate, which can be defined by the formula of *G* = *α*_c_ *R*, where *G* is the growth rate; *α*_c_ is the adhesion coefficient; and *R* is the deposition rate.^[^ ^100^ ^]^ The surface energy of the (*hk*1) crystal planes of the Sb_2_(S_x_Se_1−x_)_3_ film is relatively high, resulting in a high growth rate along this orientation and a high adhesion coefficient.^[^ ^117^ ^]^ On the contrary, the vdW bonding strength along the (*hk*0) crystal plane is weak, resulting in low adhesion to the substrate and a slow growth rate along this direction. When the vapor particles reach the substrate surface with higher kinetic energy, atoms can be rearranged on the substrate surface driven by sufficient energy, making Sb_2_(S_x_Se_1−x_)_3_ film tend to expose high‐energy (*hk*1) crystal planes.^[^ ^94^ ^]^ In other words, increasing the kinetic energy of the deposited particles is beneficial for obtaining highly oriented Sb_2_(S_x_Se_1−x_)_3_ films, which has been demonstrated by multiple groups.

#### 4.1.1 Particle Kinetic Energy

For the vacuum deposition methods (e.g., coevaporation,^[^ ^81^ ^]^ RTE,^[^ ^6^ ^]^ CSS,^[^ ^118^ ^]^ VTD,^[^ ^74^, ^75^ ^]^ magnetron sputtering,^[^ ^92^, ^119^ ^]^ and pulsed laser deposition),^[^ ^120^ ^]^ the strategies developed for regulating the vapor particle kinetic energy (e.g., adjustment of the evaporation source temperature,^[^ ^74^, ^75^, ^100^ ^]^ source–substrate distance,^[^ ^6^, ^74^, ^81^ ^]^ vapor concentration,^[^ ^99^, ^101^ ^]^ and chamber pressure)^[^ ^86^ ^]^ demonstrate that an increase in the kinetic energy of the vapor particles can effectively increase the [*hk*1] orientation of the Sb_2_(S_x_Se_1−x_)_3_ films.

A similar trend is observed in the solution methods for preparing Sb_2_(S_x_Se_1−x_)_3_ films. For example, the rapid hydrothermal deposition is kinetically more favorable to the vertical growth of (Sb_4_S(e)_6_)_n_ ribbons. Tang et al. prepared Sb_2_(S_x_Se_1−x_)_3_ films using the hydrothermal method. They found that increasing the selenourea ratio in the precursor solution accelerated the film growth and enhanced the [*hk*1] orientation, enabling the efficiency of the Sb_2_(S_x_Se_1−x_)_3_ solar cells to exceed 10% for the first time.^[^ ^36^ ^]^ Liu et al. introduced thiourea into the precursor solution of KSbC_4_H_4_O_7_·0.5H_2_O and Na_2_SeSO_3_ to complex SbO^+^ ions in the Sb_2_Se_3_ deposition to inhibit the generation of the Sb_2_O_3_ precipitation and promote the Sb_2_Se_3_ film growth rate. They prepared an enhanced [*hk*1]‐oriented film, with the corresponding device showing a 7.9% efficiency.^[^ ^38^ ^]^ Meanwhile, Zhao et al. introduced selenourea as both a complexing agent and a selenium source to the same precursor solution, further promoting the growth rate of Sb_2_Se_3_ films using the CBD method. They prepared Sb_2_Se_3_ films with [*hk*1]‐preferred orientations, eventually obtaining a 10.57% PCE, which was the highest Sb_2_Se_3_ solar cell efficiency at that time.^[^ ^45^ ^]^ Wang et al. used the CBD method and combined different sulfur sources (i.e., Na_2_S_2_O_3_ and C_2_H_5_NS) to accelerate the release rate of sulfur ions and the growth rate of Sb_2_S_3_ films. These enhanced the growth of the films and their [*hk*1] orientation, resulting in the currently highest PCE of 8% for Sb_2_S_3_ solar cells.^[^ ^48^ ^]^

The obtained results proved that increasing the kinetic energy of the ions deposited in the vacuum or solution deposition system can effectively enhance the [*hk*1] orientation of the thin film, thereby also improving the solar cell device performance. However, particles with excessive energy are detrimental to the film quality and the device performance because they can cause lattice displacement, generate lattice defects, decrease the film crystallinity, and produce pinholes.^[^ ^74^, ^99^, ^102^ ^]^ In addition, a further increase in the film growth rate leads to the whisker formation and secondary nuclei generation, resulting in the film growing in a random orientation.^[^ ^100^ ^]^ Therefore, aside from the particle kinetic energy, other factors (e.g., substrate temperature) must also be considered for the optimal growth of Sb_2_(S_x_Se_1−x_)_3_ films.

#### 4.1.2 Substrate Temperature

**Figure 7:**
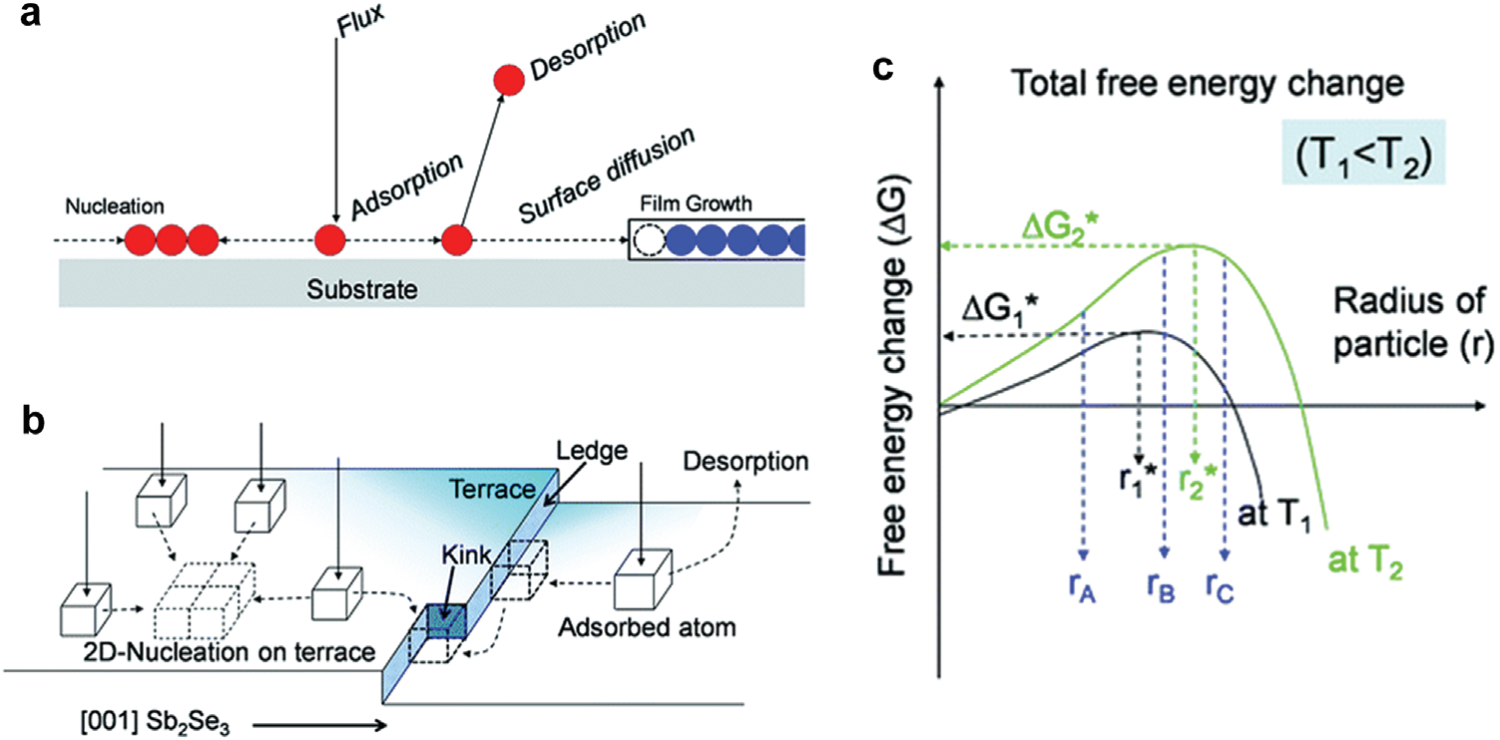
a) Nucleation and growth process during the vacuum deposition of thin films. b) Kink–ledge–terrace model for the Sb_2_Se_3_ grain growth. c) Functional curve of the total free energy versus the substrate temperature. Reproduced with permission.^[^ ^80^ ^]^ Copyright 2019, the Royal Society of Chemistry.

For the vacuum deposition method, the thin‐film nucleation and growth involve a series of kinetic processes, including the particle (atoms or molecules) deposition, re‐evaporation and migration on the substrate, and the subsequent crystal nucleation and growth processes (**Figure** **7**a). The vapor particle adsorption and migration on the crystal surface follow the terrace–ledge–kink model (Figure 7b), while the dangling bonds and the surface energy of the crystal planes increase in the order of terraces, ledges, and kinks.^[^ ^121^ ^]^ In the Sb_2_(S_x_Se_1−x_)_3_ films, the (*hk*0) crystal planes parallel to the side of the (Sb_4_S(e)_6_)_n_ ribbons have no dangling bonds. Moreover, their corresponding surface energy is low. By contrast, the (*hk*1) planes with a large number of dangling bonds have a high surface energy.^[^ ^6^ ^]^ Therefore, the (*hk*0) planes of the Sb_2_(S_x_Se_1−x_)_3_ films are likely terraces.^[^ ^80^ ^]^ Park et al. explored the effect of the substrate temperature on the film orientation based on this model. They found that the critical radius of the crystal nucleus, the potential barrier for a stable nucleus formation, the diffusion length, and desorption rate of the adsorbed atoms increase as the substrate temperature increases (Figure 7c). The density of the dangling bonds on the crystal facets increases in the order of terrace–ledge–kink, resulting in an increase in the reactivity, and enabling terrace‐adsorbed atoms to easily diffuse to the ledges and kinks. There are no dangling bonds on the (*hk*0) crystal planes, and their surface energy is low. Thus, the adsorbed atoms will diffuse onto the (*hk*1) planes and lead to crystal growth along the [*hk*1] orientation.^[^ ^80^ ^]^

**Figure 8:**
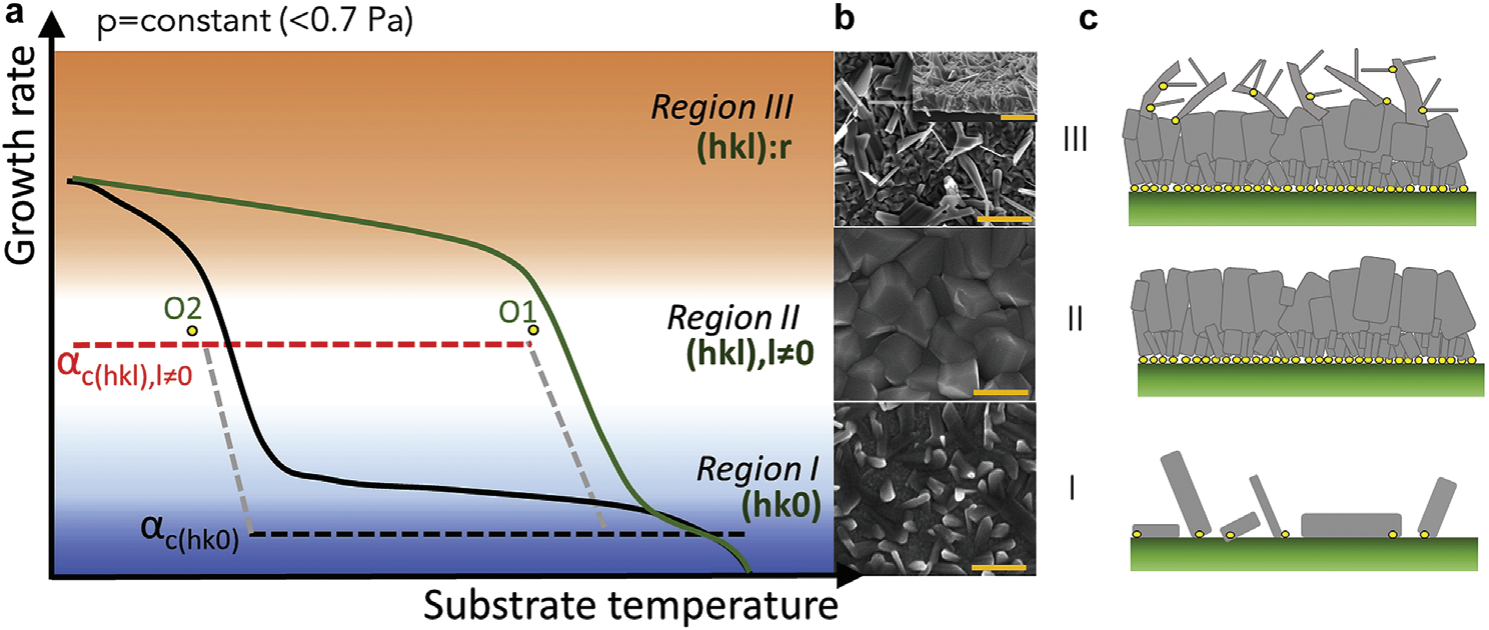
a) Growth rate as a function of the substrate temperature. α_c(_ *_hk_*_0)_ and α_c(_ *_hkl_*_,_ *_l_*_≠0)_ are the adhesion coefficients on the (*hk*0) and (*hkl*, *l*≠0) surfaces, respectively (dashed lines). b) Scanning electron microscopy images of the Sb_2_Se_3_ morphology deposited on the ZnO substrate under the three growth regimes (from top to bottom): T_sub_ = 205 °C, T_sour_  =  560 °C; T_sub_  =  243 °C, T_sour_  =  510 °C; and T_sub_  =  275 °C, T_sour_  =  510°C. c) Schematic representation of the Sb_2_Se_3_ microstructure grown under the three discussed regimes by the VTD method. The yellow dots depict the initial nuclei. Reproduced with permission.^[^ ^100^ ^]^ Copyright 2019, Elsevier.

The adhesion coefficient is related to the substrate properties determining the initial nucleus orientation. For Sb_2_(S_x_Se_1−x_)_3_, the [*hk*1]‐oriented crystal nuclei have a high adhesion coefficient to the substrate, while the [*hk*0]‐oriented nuclei have a small value. The adhesion coefficient and the growth rate of the film vary with the changes in the substrate temperature. Kondrotas et al. systematically summarized the effect of the substrate temperature on the Sb_2_(S_x_Se_1−x_)_3_ film orientation (**Figure** **8**a–c).^[^ ^100^ ^]^ The growth rate of the Sb_2_(S_x_Se_1−x_)_3_ film can be accelerated to a certain extent by increasing the evaporation temperature or decreasing the substrate temperature, consequently resulting in [*hk*1]‐oriented films. Further increasing the growth rate to Region III will lead to the whisker formation and secondary nucleation, thus resulting in a nearly randomly oriented growth. Therefore, the Sb_2_(S_x_Se_1−x_)_3_ films deposited within a narrow substrate temperature range exhibit an [*hk*1]‐preferred orientation. This temperature range varies with the substrate types. For example, using CdS, MoSe_2_, and ZnO as substrates tends to induce an [*hk*1]‐preferred orientation of the Sb_2_(S_x_Se_1−x_)_3_ films. Their most appropriate substrate temperature range is usually higher than that of the TiO_2_ and Mo substrates which tend to induce the [*hk*0] orientation.^[^ ^15^, ^20^, ^28^, ^100^, ^122^, ^123^, ^124^, ^125^ ^]^ Several groups have used the VTD,^[^ ^100^ ^]^ coevaporation,^[^ ^80^, ^81^ ^]^ and CSS^[^ ^17^ ^,100]^ techniques to deposit Sb_2_(S_x_Se_1−x_)_3_ films. These works demonstrated the dependence of the optimal substrate temperature window for obtaining [*hk*1] orientation on different substrate types.

In conclusion, both evaporation and substrate temperature affect the Sb_2_(S_x_Se_1−x_)_3_ film orientation, while a low substrate temperature leads to a decrease in the grain size and crystallinity.^[^ ^100^ ^]^ Therefore, one of the optimal growth conditions is to achieve a balanced temperature between the evaporation source and the substrate, such as obtaining Sb_2_(S_x_Se_1−x_)_3_ films with enhanced [*hk*1] orientation and high crystallinity. The above discussion provides insights on improving the deposition parameters to obtain the optimal performance of the vacuum‐deposited Sb_2_(S_x_Se_1−x_)_3_ films. Notably, due to TE has a lower evaporation rate than that of CSS, RTE, and VTD technologies, selecting a suitable substrate is more important for it to obtain highly [*hk*1]‐oriented and crystalline Sb_2_(S_x_Se_1−x_)_3_ films. We explain the mechanisms of the substrate type influence on the orientations in Section 4.3.

#### 4.1.3 Substrate Morphology

**Figure 9:**
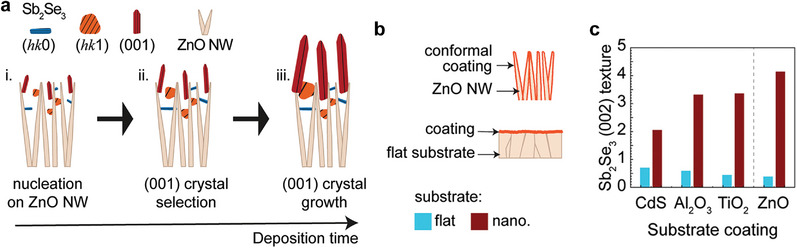
a) Mechanism of the Sb_2_Se_3_ crystal growth on the ZnO‐NW for forming highly (001) oriented films. b) Schematic of the coated nanostructured and flat substrates. c) (002) texture coefficients of Sb_2_Se_3_ films with CdS, Al_2_O_3_, and TiO_2_ coatings on flat FTO and ZnO‐NW substrates. Reproduced with permission under the terms of the Creative Commons CC‐BY‐NC‐ND license.^[^ ^18^ ^]^ Copyright 2023, Otavio Mendes et al., Wiley‐VCH.

The substrate surface morphology or flatness has a regulatory effect on the morphology and orientation of the Sb_2_(S_x_Se_1−x_)_3_ films. In this regard, Otavio Mendes et al. proposed an “orientation filtering” method for growing [001]‐oriented Sb_2_Se_3_ nanorod arrays on the ZnO nanorod array substrate. They demonstrated that the Sb_2_Se_3_ grains with [*hk*0] and low‐angle [*hk*1] orientations grow in the ZnO nanorod interstices, while the Sb_2_Se_3_ nanorods with the [001] orientation grow at the top of the ZnO nanorods (**Figure** **9**a). The degree of [001] orientation decreases with the increasing ZnO nanorod density. The authors further deposited thin CdS, Al_2_O_3_, and TiO_2_ overlayers on the ZnO nanorods, but the morphology and orientation of the grown Sb_2_Se_3_ nanorods almost did not change (Figure 9b,c). In other words, the formation of the Sb_2_Se_3_ nanorod arrays entirely depends on the substrate morphology and the growth kinetics, rather than the substrate type. This study provides a new method for preparing [001]‐oriented Sb_2_(S_x_Se_1−x_)_3_ films.^[^ ^18^ ^]^

### 4.2 Posttreatment

#### 4.2.1 Postannealing Process

**Figure 10:**
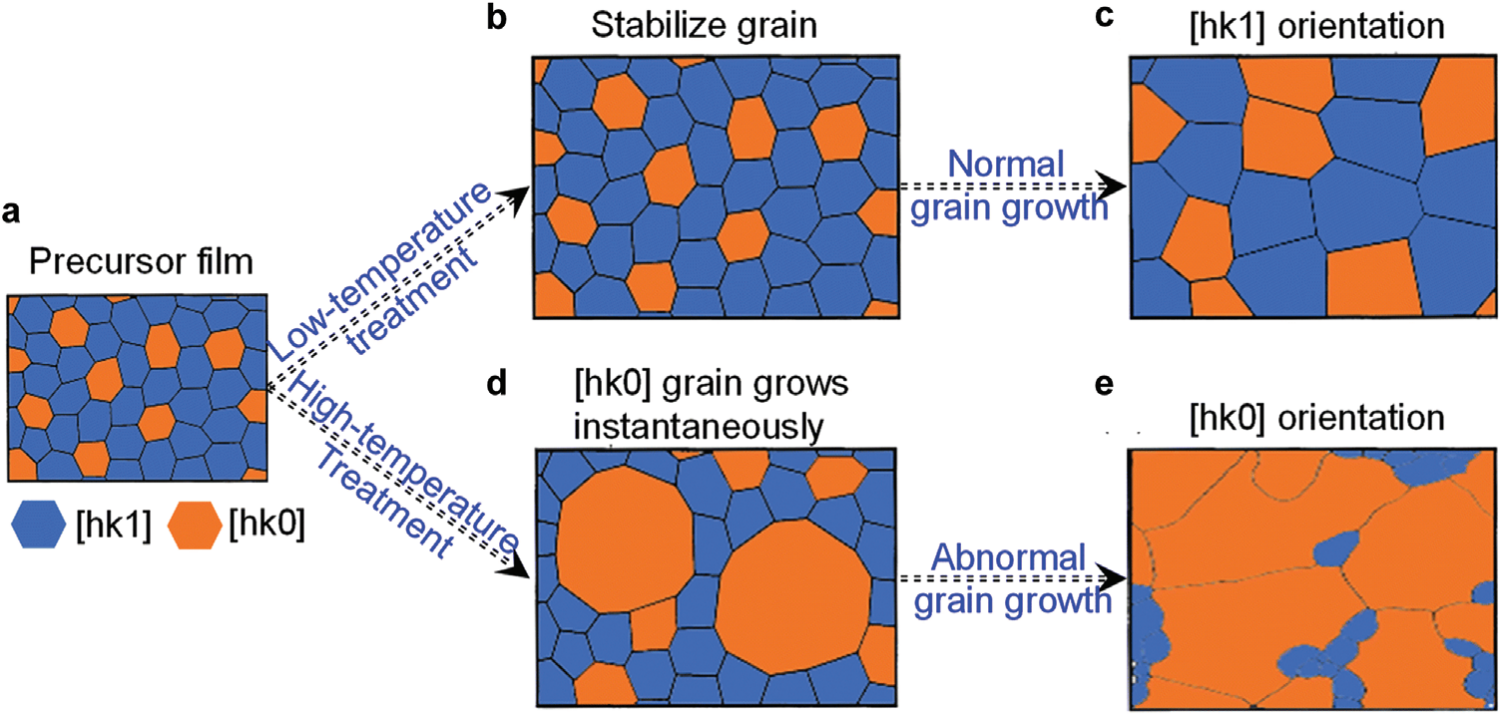
Mechanism of the Sb_2_S_3_ orientation dependence on the grain growth. Surface texture of the a) Sb_2_S_3_ precursor film, b) Sb_2_S_3_ film after low‐temperature stabilization treatment, and c) Sb_2_S_3_ film after normal grain growth. d, e) Surface texture of the Sb_2_S_3_ film undergoing an abnormal grain growth process. Reproduced with permission.^[^ ^104^ ^]^ Copyright 2023, the Royal Society of Chemistry.

Based on the principle of crystal surface energy minimization, the postannealing temperature is considered another non‐negligible factor affecting the Sb_2_(S_x_Se_1−x_)_3_ film orientation. Wu et al. investigated the influence of the annealing process on the Sb_2_S_3_ film orientation and proposed a possible crystal growth model (**Figure** **10**). Compared to the fast‐heating annealing method, the slow‐heating annealing treatment was more conducive to obtaining [*hk*1]‐oriented Sb_2_S_3_ films. They suggested that the direct high‐temperature (380 °C) annealing treatment of the precursor film can lead to abnormal grain growth, ultimately exposing more (*hk*0) crystal planes to minimize the surface energy (Figure 10a,d,e). On the contrary, when the precursor film was first treated at a low temperature of <300°C, and then heated to 380°C within a certain period, the low‐temperature treatment can stabilize the film morphology and orientation, resulting in the grains growing in a relatively uniform manner in the subsequent high‐temperature annealing, and can maintain the same morphology and orientation as the precursor. The orientation of the hydrothermally deposited Sb_2_S_3_ precursor film was more inclined toward [*hk*1] (Figure 10a–c), such that the slow‐heating annealing process can increase the [*hk*1]‐preferred orientation of the Sb_2_S_3_ films.^[^ ^104^ ^]^

In addition to the heating process, Yuan et al. found that the cooling process also affects the crystal orientation of the Sb_2_S_3_ films. Through a rapid cooling treatment of the RTE‐deposited Sb_2_S_3_ film, the (200) crystal plane growth was found suppressed, resulting in better electron transport and a device performance improvement.^[^ ^126^ ^]^ Zhang et al. found that the preferred Sb_2_Se_3_ orientation varies with the annealing atmospheres. The Sb_2_Se_3_ films annealed in the H_2_S and H_2_Se atmospheres tend to expose (020) and (120) planes, respectively, while those annealed in an inert Ar atmosphere tend to expose (211) and (221) planes.^[^ ^78^ ^]^ Li et al. demonstrated that the posttreatment of Sb_2_(S_x_Se_1−x_)_3_ films with potassium iodide in a vacuum annealing environment can enhance the [211] orientation.^[^ ^103^ ^]^

#### 4.2.2 Selenization Kinetics

A postselenization approach that allows the Sb_2_Se_3_ films to exhibit a [*hk*1]‐oriented growth from the top to the substrate has been presented.^[^ ^12^, ^13^, ^86^, ^87^, ^88^, ^89^, ^90^ ^]^ In a tube furnace, when treating the Sb film deposited on the substrate using the Se vapor generated by the Se powder, the diffusion and selenization reactions occur as Se atoms reach the Sb film surface. The selenization reaction is faster than the diffusion reaction; hence, the formed (Sb_4_Se_6_)_n_ ribbon allows the Se atoms to resist the vdW forces and continuously undergo selenization downward along the [001] direction. This results in Sb_2_Se_3_ films with ribbons perpendicular to the substrate.^[^ ^13^ ^]^ The orientation regulation through the postselenization treatment on the absorber layer is mainly driven by the selenization kinetics. Notably, the [001]‐oriented growth can be controlled by balancing the collision rate and the kinetic energy of the Se vapor particles reaching the film surface. Based on this, Wen et al. prepared [001]‐oriented Sb_2_Se_3_ films by controlling the vapor pressure to regulate the collision rate and the kinetic energy of the Se vapor particles reaching the Sb film surface. They obtained an efficiency of 8.42% for the flexible Sb_2_Se_3_ devices.^[^ ^86^ ^]^ The orientation control is driven by selenization kinetics; therefore, the substrate type is no longer the main factor affecting the film orientation. Zhou et al. conducted a postselenization treatment on the Sb_2_Se_3_ films deposited on MoSe_2_, soda lime glass (SLG), NiO, SiO_2_, and FTO substrates. All the treated films exhibited a [001] orientation consistent with the above‐mentioned conclusions.^[^ ^13^ ^]^

During the postselenization process, the high‐energy Se vapor may considerably affect the behavior of the adsorbed atoms and the nucleus–substrate interaction, consequently altering the film growth kinetics with specific morphology and orientation. Under the Se atmosphere, the (*hk*1) crystal plane will have a higher growth rate due to the increased energy of the adsorbed atoms, thereby forming preferred [*hk*1] orientation. Liang et al. and Fan et al. successfully transformed the [*hk*0] orientation of the Sb_2_Se_3_ films deposited through VTD^[^ ^76^ ^]^ and CSS^[^ ^20^ ^]^ methods into [*hk*1] orientation through the postselenization treatment. Wang et al. prepared Sb_2_S_3_ films on FTO/CdS substrates through TE method and demonstrated the positive effect of the postselenization treatment on the [*hk*1] orientation enhancement.^[^ ^127^ ^]^ Mavlonov et al. developed a PLD in situ selenization technique for preparing Sb_2_Se_3_ films. They achieved an [*hk*1]‐preferred orientation by adjusting the Sb:Se atomic ratio of the target material to 1:3 to render a Se‐rich state.^[^ ^120^ ^]^ Peng et al. prepared Sb_2_S_3_ films with preferred [*hk*1] orientation by post‐sulfurizing the Sb films that were deposited on the Mo substrates using pulse electrodeposition. They obtained the highest efficiency of 3.35% for the substrate‐structure Sb_2_S_3_ solar cells.^[^ ^10^ ^]^

In addition to vapor selenization, an effective liquid selenization method has recently been proposed. Yao et al. developed a liquid medium annealing (LMA) method to regulate the Sb_2_S_3_ orientation by controlling the Se_2_^2−^ concentration in the Se_2_^2−^–oleylamine complex. Due to the high thermal conductivity and high Se activity in the LMA system, the activated Se_2_^2−^ anions can react with Sb_2_S_3_ to activate the apical bud growth on the (Sb_4_S_6_)_n_ ribbons, along with releasing the oleylamine adsorbed on the (*hk*0) plane to facilitate the growth of the [*hk*1] orientation. In this process, the bottom of the Sb_2_S_3_ layer undergoes recrystallization to inherit the [*hk*1] orientation of the Sb_2_(S_x_Se_1−x_)_3_ seeding material at the top, ultimately obtaining a highly oriented Sb_2_(S_x_Se_1−x_)_3_ film.^[^ ^3^ ^]^

### 4.3 Substrate Type

**Figure 11:**
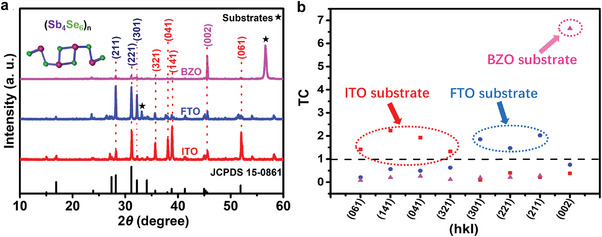
a) XRD patterns and b) texture coefficients of the diffraction peaks of the Sb_2_Se_3_ thin films deposited on the ITO, FTO, and BZO substrates. Reproduced with permission.^[^ ^21^ ^]^ Copyright 2021, Elsevier.

The Sb_2_(S_x_Se_1−x_)_3_ film orientation is strongly dependent on the substrate type. Zhou et al. investigated the effect of the indium‐doped tin oxide (ITO), fluorine‐doped tin oxide (FTO), and boron‐doped zinc oxide (BZO) substrates on the orientation of the CSS‐Sb_2_Se_3_ films.^[^ ^21^ ^]^ **Figure** **11** presents the results. The Sb_2_Se_3_ film grown on ITO mainly exhibited an [*hk*0] orientation, while that grown on FTO mainly showed [211] and [221] orientations, indicating that the ribbons grown on FTO were more perpendicular to the substrate than those on ITO. Notably, the Sb_2_Se_3_ film deposited on BZO exhibited a highly oriented growth with a distinct (002) crystal plane exposed, indicating that the ribbons grew to be completely perpendicular to the substrate. B doping in BZO can induce the lattice deformation of ZnO, thereby increasing the exposure proportion of nonpolar (110) crystal planes.^[^ ^128^, ^129^ ^]^ Cao et al. prepared Sb_2_Se_3_ nanorod array structured films with a [001] orientation on a BZO substrate.^[^ ^128^ ^]^

Spaggiari et al. investigated the growth orientation of the Sb_2_Se_3_ films deposited by RF sputtering method on SLG, FTO, Mo, ZnO, and CdS substrates.^[^ ^27^ ^]^ They demonstrated that the Sb_2_Se_3_ films grown on the CdS and ZnO substrates exhibit superior vertical orientation, while those grown on Mo substrates have the poorest vertical orientation. Kondrotas et al.^[^ ^100^ ^]^ and Zeng et al.^[^ ^71^ ^]^ drew a similar conclusion using the VTD and RTE methods. Valdman et al. demonstrated the epitaxial growth of the [001]‐oriented (Sb_4_Se_6_)_n_ ribbons on the [100]‐oriented mica substrate.^[^ ^130^ ^]^ Wen et al. demonstrated that the CdS grown on mica substrates can induce the growth of [*hk*1]‐oriented Sb_2_Se_3_ films. As a result, a flexible Sb_2_Se_3_ solar cell based on the mica substrate achieved a high efficiency of 7.15%.^[^ ^14^ ^]^

Tang et al. analyzed the mechanism of the effect of substrate types on the growth orientation of Sb_2_(S_x_Se_1−x_)_3_ films from the bonding energy perspective. The bonding energy of Ti─O (662 kJ mol^−1^) is much stronger than that of Cd–S (196 kJ mol^−1^) and Zn─O (284 kJ mol^−1^); thus, the Sb and Se atoms can hardly bond to the TiO_2_ substrate during the film deposition. This results in most (Sb_4_S(e)_6_)_n_ ribbons lying lateral on the substrate and forming [*hk*0]‐oriented Sb_2_(S_x_Se_1−x_)_3_ films.^[^ ^8^ ^]^ According to the lattice matching mechanism, Deng et al. found that the exposed (101) crystal planes of TiO_2_ particle films are favorable to Sb_2_S_3_ film growing along the [*hk*1] orientation.^[^ ^61^ ^]^ Dong et al. compared the orientations of the hydrothermally deposited Sb_2_(S_x_Se_1−x_)_3_ films grown on crystalline and amorphous TiO_2_ nanoparticle films. They found that the film is more likely to nucleation on amorphous TiO_2_ and grow along the [*hk*1]‐preferred orientation by alleviating the lattice mismatch between the amorphous TiO_2_ and the [*hk*1]‐oriented Sb_2_(S_x_Se_1−x_)_3_ film. From the crystal surface energy perspective, Wang et al. demonstrated that the low‐energy SnO_2_ (110) or TiO_2_ (101) facets both tend to coordinate with the Se and Sb atoms, inducing (Sb_4_Se_6_)_n_ ribbons to grow along the [*hk*1] orientation.^[^ ^105^ ^]^

### 4.4 Interfacial Engineering

Feasible strategies have been proposed to regulate the crystal plane exposure to improve the lattice matching degree between the interface material and the [*hk*1]‐oriented (Sb_4_S(e)_6_)_n_ ribbons. A common method for such a purpose is to selectively control the nucleation and growth rates in different directions during crystal growth. Some inorganic or organic additives have been used as the capping agents for selectively adsorbing on the specific crystal planes, thereby regulating the crystal plane exposure.^[^ ^131^ ^]^ According to the crystal growth theory, the growth rate of the specific crystal planes can be evaluated using surface energies. For CdS, the polar planes with large surface energies (e.g., (101) and (002)) will exhibit high growth rates during the film growth and quickly disappear from the crystal surface, eventually exposing more nonpolar crystal planes (e.g., (100) planes). The Sb and S(e) atoms in the Sb_2_(S_x_Se_1−x_)_3_ precursor films tend to form a chemical bond with S and Cd on the nonpolar (100) plane of CdS, which is favorable for the (Sb_4_S(e)_6_)_n_ ribbons growing along the [*hk*1] direction. In contrast, the S or Cd atoms alternatively distributed on the polar (002) plane of CdS preferentially coordinate with Sb or S(e) atoms of the (Sb_4_S(e)_6_)_n_ ribbons through van der Waals face, enabling the ribbons tend to grow parallel to the substrate.^[^ ^81^ ^]^

Based on this theory, Jin et al. regulated the exposed planes of CdS films by adding Cd ions to the hydrothermal precursor solution to enhance the [*hk*1]‐oriented growth of Sb_2_(S_x_Se_1−x_)_3_ films and ultimately obtained the enhanced device performance.^[^ ^11^, ^41^ ^]^ Yin et al. reported that the exposed crystal planes of CdS can be adjusted by tuning the sequence of the coevaporation order of S and Sb_2_Se_3_, leading to the (Sb_4_S(e)_6_)_n_ ribbons growing vertically on the CdS substrate and exhibiting an enhancement of the [*hk*1] orientation.^[^ ^81^ ^]^ Li et al. prepared CdS:O films through a molecular beam epitaxial deposition method, and proposed that O atoms have distinct interactions on different crystal planes of CdS. Based on the density functional theory calculations, the O doping in CdS is demonstrated to increase the exposure of nonpolar (100) plane and enhance the [*hk*1] orientation of (Sb_4_S(e)_6_)_n_ ribbons.^[^ ^106^ ^]^ Pan et al. prepared CdS with an exposed (103) crystal plane using the CSS method. They then demonstrated that this exposed facet can induce the vertical anchoring of the (Sb_4_S(e)_6_)_n_ ribbons prepared by VTD method.^[^ ^73^ ^]^

Fluoride ions (F^−^) are widely used as capping agents for regulating the growth rate and exposure of the crystal planes due to their unique selective adsorption properties.^[^ ^132^ ^]^ Shi et al. introduced F^−^ into the hydrothermal precursor solution. They proposed that the F^−^ could preferentially adsorb on the (101) crystal plane of CdS, increasing the exposure of nonpolar (100) plane of CdS and promoting the [*hk*1]‐preferred orientation of Sb_2_(S_x_Se_1−x_)_3_. F^−^ can also selectively adsorb on the side of the (Sb_4_S(e)_6_)_n_ ribbons, thereby hindering the lateral growth of the ribbons and promoting their longitudinal growth.^[^ ^42^ ^]^ Chloride ions and carboxylate anions were demonstrated to have similar effects according to Chen et al.^[^ ^43^ ^]^ and Yang et al.^[^ ^54^ ^]^ Based on the high mobility and activity of lithium ions, Mao et al. proposed a strategy for annealing Sb_2_S_3_ films in a LiCl molten salt bath. They successfully doped Li into Sb_2_S_3_ while enhancing the [*hk*1] orientation. As a result, they achieved a 6.16% PCE, which was the highest value among all inorganic Sb_2_S_3_ solar cells at that time.^[^ ^40^ ^]^ They also found that Bi doping can exhibit a similar effect to Li.^[^ ^39^ ^]^

As mentioned above, the Mo substrate is not favorable for the [*hk*1]‐oriented growth of the (Sb_4_S(e)_6_)_n_ ribbons. By performing a postselenization treatment on the Mo substrate to form a MoSe_2_ interfacial layer, Li et al. prepared vertically oriented Sb_2_Se_3_ nanorod array films through the CSS and injection vapor deposition (IVD) methods. Using these methods, they obtained the top efficiencies of 9.2% and 10.1%, respectively, for the Sb_2_Se_3_ solar cells.^[^ ^17^, ^45^ ^]^ Similarly, Liang et al. introduced a WSe_2_ layer by selenizing the W back‐contact layer, which reduced the interfacial lattice mismatch with the (Sb_4_Se_6_)_n_ ribbons. They then prepared [001]‐oriented Sb_2_Se_3_ nanorod arrays on WSe_2_ using the CSS method.^[^ ^67^ ^]^ Subsequently, they evaporated a thin PbSe layer at the buried back‐contact interface on the Mo foil to promote the growth of [*hk*1]‐oriented Sb_2_Se_3_ films. As a result, they obtained the highest PCE of 8.43% for the flexible Sb_2_Se_3_ solar cells.^[^ ^7^ ^]^

Note that the surface state of the substrate may also vary during the vacuum deposition process, thereby influencing the orientation of subsequently deposited films. Due to the high saturated vapor pressure of S, the S loss will inevitably occur during the vacuum deposition of CdS films. This will lead to an increase in the Cd^2+^ dangling bonds on the CdS surface and ultimately result in different bonding modes with the Sb_2_(S_x_Se_1−x_)_3_ films. Zeng et al. demonstrated the above conclusion by comparing the different orientation characteristics of Sb_2_S_3_ films deposited on CdS substrates using RTE and VTD vacuum methods. The CdS surface was easily surrounded by saturated S vapor due to the closer source–substrate distance of the RTE, resulting in a saturated S state on the film surface. Consequently, only one type of bonding was formed between the (Sb_4_S_6_)_n_ ribbons and the CdS surface. This led the ribbons to be parallel to the CdS substrate. However, the source–substrate distance in VTD is relatively large, and S loss is prone to occur during the film deposition. This results in an increase in the Cd dangling bonds on the CdS surface, which is more conducive for obtaining vertically oriented Sb_2_S_3_ films.^[^ ^70^ ^]^

Researchers have also proposed effective interfacial modification strategies for regulating the absorber layer orientation. The high bonding energy of Sn─O and Ti─O hinders the vertical growth of (Sb_4_S(e)_6_)_n_ ribbons on SnO_2_ and TiO_2_ substrates. Based on this issue, Zhou et al. used CdCl_2_ to treat the SnO_2_ substrate and form a Cd─O bond with bonding energy much lower than Sn─O, thereby inducing the vertical growth of (Sb_4_S_6_)_n_ ribbons.^[^ ^68^ ^]^ Meanwhile, Wang et al. treated the TiO_2_ substrates using TiCl_4_, consequently enhancing the interfacial bonding between the TiO_2_ and Sb_2_Se_3_ absorber layers with the introduction of Cl atoms, and promoting the vertical growth of (Sb_4_Se_6_)_n_ ribbons.^[^ ^110^ ^]^ Cai et al. used SbCl_3_ to modify the CdS surface, thereby increasing the exposed nonpolar planes of CdS and enhancing the [*hk*1]‐oriented Sb_2_Se_3_ films.^[^ ^82^ ^]^ Wang et al. treated the CdS surface with ammonia, enhancing the [*hk*1] orientation of Sb_2_Se_3_ films, as well.^[^ ^109^ ^]^ Guo L. et al. prepared CdS:O films by postannealing the CdS at different oxygen partial pressures. They demonstrated that oxygen doping could significantly improve the [*hk*1] orientation of Sb_2_Se_3_ and illustrated that the improved orientation can be attributed to the O diffusion between the (Sb_4_Se_6_)_n_ ribbons, forming Sb─O─Se chains and changing the growth orientation of the (Sb_4_Se_6_)_n_ ribbons.^[^ ^108^ ^]^ Furthermore, Guo H. et al. treated the CdS surface with oxygen plasma and demonstrated the beneficial effect of the increased oxygen content on CdS surface on the vertical growth of the Sb_2_Se_3_ films.^[^ ^133^ ^]^

### 4.5 Seeding Material

According to the film growth theory, the initial seed determines the final film orientation.^[^ ^8^, ^104^, ^114^ ^]^ Eliminating dominant [*hk*0]‐oriented seeds and retaining [*hk*1]‐oriented seeds are the key steps to obtain [*hk*1]‐oriented Sb_2_(S_x_Se_1−x_)_3_ films. Due to the weak vdW force that binds the [*hk*0]‐oriented seeds to the substrate, adjusting the substrate heating method can break the adhesion between them.^[^ ^8^ ^]^ Sb_2_S_3_ and Sb_2_Se_3_ have the same crystal structure; hence, they are often employed as the seeding materials for the oriented growth of the Sb_2_(S_x_Se_1−x_)_3_ films.^[^ ^8^, ^15^, ^69^, ^113^, ^114^, ^134^ ^]^ Some metals^[^ ^135^ ^]^ and metal oxides^[^ ^58^, ^111^, ^112^, ^115^ ^]^ have also been used as crystal seeding materials for regulating the Sb_2_(S_x_Se_1−x_)_3_ film orientation.

**Figure 12:**
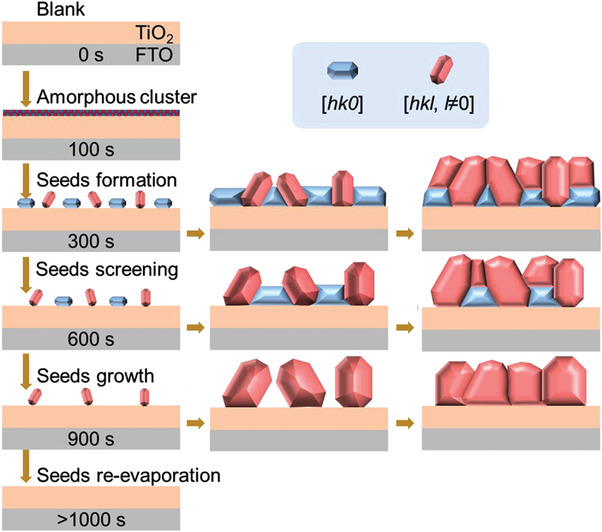
Growth model of the Sb_2_Se_3_ seed evolution with duration time (left section), Sb_2_Se_3_ film growth based on the corresponding seeds (middle section), and Sb_2_Se_3_ films with an increased thickness (right section). Reproduced with permission.^[^ ^8^ ^]^ Copyright 2021, Wiley‐VCH.

Li et al. proposed a “seed screening” strategy for regulating the Sb_2_Se_3_ orientation based on the different bonding strengths of the [*hk*0]‐ and [*hk*1]‐oriented (Sb_4_Se_6_)_n_ ribbons with the substrate. In **Figure** **12**, a gradient heating method was used to recrystallize the seeding material at high temperatures, thereby removing the [*hk*0]‐oriented Sb_2_Se_3_ seeds deposited at the low temperatures and retaining the [*hk*1]‐oriented seeding material. The subsequently deposited Sb_2_Se_3_ films grew along the [*hk*1] orientation induced by the seeding material. This regulation method was fundamentally different from the previously mentioned approach of regulating the substrate temperature to control the growth orientation. This technique achieved a 7.62% PCE, which is currently the highest efficiency of the Sb_2_Se_3_ solar cells based on TiO_2_ substrates.^[^ ^8^ ^]^ Spalatu and Krautmann et al. enhanced the [001] orientation of the subsequently deposited Sb_2_Se_3_ films by predepositing a thin Sb_2_Se_3_ seeding material on the TiO_2_ and CdS substrates using CSS method.^[^ ^15^, ^114^ ^]^ Amin et al. improved the vertical orientation of subsequently CSS‐deposited Sb_2_Se_3_ films by using hydrothermally deposited Sb_2_S_3_ as the seeding material on CdS.^[^ ^113^ ^]^ Rijal et al. used pre‐prepared [*hk*1]‐oriented Sb_2_Se_3_ as the seeding material and prepared highly [*hk*1]‐oriented Sb_2_Se_3_ films on Mo substrate through CSS method, achieving a PCE of 8.5% for the substrate‐structure solar cells.^[^ ^69^ ^]^

The spatial distance of Ti─O (4.71 nm) on the (101) plane of TiO_2_ actually matched well with the spatial distance of S–Sb (4.79 nm) on the (101) plane of Sb_2_S_3_. Thus, a good lattice matching was found between the orthotropic [101]‐oriented Sb_2_S_3_ and the anatase [101]‐oriented TiO_2_. Wang et al. developed a seed‐assisted solution method for the pre‐spin coated [*hk*1]‐oriented Sb_2_S_3_ seeding material on a polycrystalline TiO_2_ nanoparticle film and prepared [*hk*1]‐preferred oriented Sb_2_S_3_^[^ ^57^, ^58^ ^]^ and Sb_2_Se_3_^[^ ^59^ ^]^ nanorod array films by repeating the spin‐coating and annealing processes. Based on the above work, Liu et al. prepared [211]–oriented Sb_2_S_3_ nanorod array films with Sb_2_Se_3_ seeding material. Unlike the Sb_2_S_3_ seeding material, which can only induce an [*hk*1]‐oriented growth on the TiO_2_ nanoparticle films, the Sb_2_Se_3_ seeding material can induce the growth of [*hk*1]‐oriented Sb_2_S_3_ films on any substrate.^[^ ^111^ ^]^

In addition to Sb_2_S_3_ and Sb_2_Se_3_, some metal oxides can also serve as seeds for inducing the oriented growth of the films. Wang et al. deposited a thin CeO_2_ layer on the CdS surface, which resulted in a smoother surface that induced the vertical growth of the Sb_2_Se_3_ grains.^[^ ^112^ ^]^ Zi et al. sputtered an Al_2_O_3_ layer between the CdS and Sb_2_Se_3_ layers, effectively inhibiting the growth of the [*hk*0]‐oriented (Sb_4_Se_6_)_n_ ribbons and promoting the [*hk*1]‐oriented growth.^[^ ^115^ ^]^ Lin et al. inserted a MoO_2_ layer between the Mo substrate and the Sb_2_Se_3_ film, consequently promoting the growth of [211]‐oriented (Sb_4_Se_6_)_n_ ribbons.^[^ ^116^ ^]^ Yang et al. demonstrated the introduction of an additional metal element to serve as a crystal seed for the oriented film preparation. They prepared an Ag:Sb_2_S_3_ thin film by sulfurizing the Ag/Sb metal precursor film predeposited on the Mo substrate and applied it as the photocathode. They proved that some AgSbS_2_ crystal units were formed in the Ag/Sb bimetallic films during the sulfurization process, which served as the [*hk*1]‐oriented seeding material that promoted the preparation of completely [*hk*1]‐oriented Sb_2_S_3_ films. This strategy can be extended to the fabrication of high‐efficiency Sb_2_S_3_ solar cells.^[^ ^135^ ^]^

## 5 Summary and Outlook

In summary, this study first outlined the main factors affecting the orientation of the Q1D Sb_2_(S_x_Se_1−x_)_3_ films and the influence of this orientation on the performance of the corresponding photovoltaic devices from the perspective of crystal orientation engineering. We summarized herein the developed strategies for improving [*hk*1]‐oriented films based on the solution and vacuum deposition methods and thoroughly explained the oriented growth mechanism. The following results were drawn in this work:

1. The crystal orientation of the Sb_2_(S_x_Se_1−x_)_3_ films is mainly influenced by the growth rate. The growth rate in the vacuum deposition methods is mainly influenced by the particle kinetic energy, the growth temperature, and the substrate properties. High‐kinetic energy particles are favorable for the growth of the [*hk*1]‐oriented Sb_2_(S_x_Se_1−x_)_3_ films, while an excessively high growth temperature is unfavorable to the [*hk*1] orientation. The substrate type can affect the film growth rate and the orientation. Sb_2_(S_x_Se_1−x_)_3_ films with a preferential orientation and a high crystallinity can be obtained by combining the abovementioned factors. In addition, different postannealing modes can affect the Sb_2_(S_x_Se_1−x_)_3_ film orientation. The postselenization treatment of the Sb thin films is a top‐down method for preparing [001]‐oriented Sb_2_Se_3_ films, which is mainly influenced by the selenization kinetics, rather than the substrate properties. Introducing additives into the precursor solution of the solution method can effectively regulate the oriented growth of the Sb_2_(S_x_Se_1−x_)_3_ film. On the one hand, the additives play an important role in regulating the particle deposition rate to improve the film orientation. On the other hand, they can act as capping agents to adsorb on specific crystalline planes to control their growth rate, thereby regulating the orientation of the (Sb_4_S(e)_6_)_n_ ribbons.
2. Interfacial lattice matching regulation is an effective method of controlling the Sb_2_(S_x_Se_1−x_)_3_ film orientation. The lattice mismatch between the Sb_2_(S_x_Se_1−x_)_3_ films and the interfacial materials of the substrate leads to a weak bonding strength of the ribbons to the substrate, which results in the ribbons tending to lay flat on the substrate to minimize the surface energy. Effective interfacial engineering strategies for interfacial materials (e.g., ion selective adsorption and interface treatment for increasing the exposure of nonpolar crystal planes) should be considered to enhance the bonding between the interfacial material and the [*hk*1]‐oriented (Sb_4_S(e)_6_)_n_ ribbons. Introducing a seeding material between the substrate and the Sb_2_(S_x_Se_1−x_)_3_ film can also enhance their lattice matching, thereby inducing the growth of [*hk*1]‐oriented ribbons.

Many feasible methods are currently being developed in the field of crystal orientation engineering. However, the following critical issues must be addressed:

1. The current highest efficiency of Sb_2_(S_x_Se_1−x_)_3_ solar cells is obtained through the hydrothermal deposition method. However, the film orientation and crystallinity prepared through this method must be further improved compared to those prepared by the vacuum deposition method. Therefore, improving the crystallinity and the [*hk*1] orientation of the hydrothermally deposited Sb_2_(S_x_Se_1−x_)_3_ films is expected to further improve the device efficiency.
2. The ways of regulating the optimal coordination between the [001]‐oriented nanorod arrays and the lateral grown [*hk*0]‐oriented grains in the films to simultaneously improve the preferred orientation, compactness, and large grain size of the Sb_2_(S_x_Se_1−x_)_3_ films is a key issue in developing 1D material‐based photovoltaic devices.
3. The existence of dislocations in the Sb_2_(S_x_Se_1−x_)_3_ films can act as defects to inhibit the carrier transport along the (Sb_4_S(e)_6_)_n_ ribbons. The dislocation formation may be related to the strains generated during the grain growth. Therefore, the development of an advanced growth process that can better control the strain is a promising direction for suppressing these internal dislocations.

## Conflict of Interest

The authors declare no conflict of interest.
